# HTM-MDICE: a transformer-based model for predicting student engagement and ideological understanding in ethical education

**DOI:** 10.3389/fpsyg.2025.1643076

**Published:** 2025-12-17

**Authors:** Chang Qin

**Affiliations:** School of Marxism, Shandong Sport University, Jinan, China

**Keywords:** individualized learning, ethical education, transformer model, predictive modeling, student

## Abstract

Tailoring individualized learning experiences depends on predicting student involvement and ideological awareness in ethical education, which is still difficult given educational data sets' complexity and class imbalance. HTM-MDICE, a new Transformer-based model meant to solve these issues by using hierarchical temporal modeling on a multi-modal ethical dataset of 68,200 scenarios, 1,000,000 numerical data points, and 500,000 behavioral logs, is presented in this paper. HTM-MDICE, utilizing a thorough evaluation framework, obtained a validation accuracy of 97.5%, an F1-score of 0.96, and an MAE of 0.12 with an early stopping patience of 5, therefore greatly outperforming four previous techniques—BSA-ANN, Decision Tree, BPNN, Petri Nets 10.5% in accuracy (*p* < 0.05). While preprocessing, early stopping, and the Transformer design were shown to be major factors in HTM-MDICE's performance, statistical analysis using paired *t*-tests verified the strength of its enhancements. Though it has improved, ethical issues around misclassification and data privacy call for prudent use. With future goals comprising improved interpretability, varied data integration, and longitudinal effect studies to further promote individualized education, this study adds a state-of-the-art model and assessment approach to educational predictive modeling.

## Introduction

Artificial intelligence (AI) was a promise and a threat in the late 1950s based on having an agent with high power that would simplify common tasks but assert its decision-making control over humans. As an autonomous, adaptive, and interactive human-made software system, AI is now capable of making decisions in complex situations based on perception, interpretation, and reasoning from data (Dignum, 2021). AI in Education (AIED) is being applied to administration to monitor the goals of the school, in accordance with policies, and to follow up on students' interests; it assists teachers in their daily tasks and facilitates lifelong learning (Miao et al., 2021). Promises of these systems include discovery and development of talents and competencies, workload release for educators, addressing student diversity, prediction of student and institutional underachievement, facilitation of transition to professional life, provision of cheaper and better-quality education to poorer students, and more effective learning experiences. This can be attributed to the COVID-19 pandemic since discussions about using these resources for online assessment support and the experiences of students are on the increase (García-Peñalvo et al., 2020). For example, a study that was conducted in Romania revealed that due to learning needs that COVID-19 Pandemic caused the use of AI-supported platforms for both teachers and learners increased within the period between 2019 and 2020 even within less developed geographies (Pantelimon et al., 2021).

Many recent papers have examined how AI might be applied in Ideological and Political Education (IPE). Every study enhances learning and teaching using a different set of techniques. The first study employed a Binary Search Algorithm (BSA) and an Artificial Neural Network (ANN) to investigate how IPE teaching and learning occur (Petrosov et al., 2021). Claimed to be 95% accurate, the approach was compared against statistical learning techniques and conventional classroom instruction. The second study examined a mixed teaching approach combining IPE philosophy with machine learning concepts. A decision tree algorithm accomplished this; student polls gauged its efficacy (Topîrceanu and Grosseck, 2017). In the third study, the paper uses a backpropagation neural network (BPNN) model to predict learning interruptions (Li et al., 2022). The fourth study simulated the online IPE process using Petri nets and discrete dynamic models (Wang and Wang, 2023). Nonlinear prediction modeling was also applied to examine how well students acquired knowledge in a large data environment.

A novel approach proposed in this paper to address the shortcomings of prior studies is the Hybrid Transformer-Based Model with Multimodal Data Integration and Human-Centric Evaluation (HTM-MDICE). The HTM-MDICE system looks at many kinds of data—text (like student essays), numbers (like grades), and behavior (like engagement logs)—using a Transformer-based design and a fine-tuned BERT model. The approach intends to accomplish two goals: correctly forecast how involved and ideologically conscious students will be and provide practical advice for teachers and students. Many modifications have been done, including thorough data preparation, 80-10-10 dataset division for training, validation, and testing, and early stopping with patience values of 3, 5, and 7 to improve performance. Multimodal data integration provides a complete picture of student learning; human-centered evaluation, backed by AI techniques that can be explained like SHAP, ensures that outcomes can be grasped. These changes are anticipated to produce better outcomes than the previous methods. The main goals of this research are: (1) to create and assess HTM-MDICE on an ethics dataset, (2) to contrast its performance with the four previous techniques using thorough metrics, and (3) to offer in-depth insights via figures and tables, including error/accuracy curves and confusion matrices. Among the contributions of this paper are the development of a strong AI model for IPE, the proof of its superiority by thorough testing, and the supply of useful tools for teachers, including tailored recommendations. This work intends to promote the use of AI in IPE by means of its shortcomings in earlier research.

In this study, we focus on Ethical Education, which refers to the process of teaching individuals how to make reasoned, reflective decisions about moral dilemmas and actions based on ethical principles. While “Moral Education” can refer more broadly to the development of moral values and virtues, Ethical Education specifically addresses decision-making processes within a framework of ethics. For clarity and consistency, we will use ‘Ethical Education' throughout the manuscript.

The paper followed a logical order, with Section 3 as the method, Section 4 as the findings, Section 5 as the discussion, and Section 6 as the conclusion.

## Related research

This section reviews the work done. Petrosov et al. (Petrosov et al., 2021) tried to enhance structural-parametric synthesis of intelligent systems by artificial neural network models for handling genetic algorithm parameters. Petri nets theory was utilized by the research to perform simulations and demonstrated its ability with CPU and CPU+GPGPU technologies.

Topîrceanu and Grosseck (Topîrceanu and Grosseck, 2017) applied data mining and supervised learning to examine profiles of students taking online courses, based on interaction type, commitment, and completion. This study provided insights for the design of eLearning.

Li et al. (2022) identified that DL-IIBEM model improved information interaction, user experience, and system performance through adding Knowledge Network Mechanism Analysis. They verified its reliability with the help of simulation analysis and ratio of performance.

Wang and Wang (2023) studied the use of big data to enhance models of ideological and political courses on university online platforms. They compared traditional teaching with formalized modeling approaches, demonstrating that dynamic modeling was able to enhance traditional teaching.

Casas-Roma et al. (2021) proposed that the integration of artificial morality tools in AI systems would enhance the effectiveness of AI decision-making in schools through the management of ethical dilemmas, particularly as the systems continue to become more autonomous.

Ghotbi and Ho (2021) conducted a survey of 467 questionnaires of Japanese and non-Japanese students at an international university, which revealed that 58% of the student's named unemployment as the most important ethical issue related to AI. The research also noted that hardly any respondents named the risks of AI.

Wang (2021) defined the potential strengths and weaknesses of AI in the data-based decision making of school leaders in relation to student efficiency and accuracy. Wang also defined the potential existence of inherent bias, moral values, and data security issues.

Zhang et al. (2022) argued that deep learning technology would refine the process of assessing ideological and moral education within universities and colleges through formative and consequential course assessment, as well as increasing the effectiveness of ideological and political education.

Akgun and Greenhow (2022) investigated the social and ethical implications of AI adoption in K-12 education. They found the applications and challenges of AI, and educational resources from MIT Media Lab and Code.org.

Leta and Vancea (2023) discussed the ethical implications of AI integration in education, promoting a model of responsible AI implementation based on robust policies and algorithm transparency.

Al-Zahrani and Alasmari (2024) examined the influence of AI on Saudi Arabian higher education, showing positive attitudes toward AI while also emphasizing the significance of ethical issues and inclusive conceptualization of AI adoption.

Han et al. (2025) Story Completion Method was employed to analyze students' fears about using AI tools to teach, revealing their ability to redefine pedagogical variables like learner autonomy and learning environments.

Abbasi et al. (2025) examined the influence of AI on the development of higher education curriculum globally, reflecting on its potential benefits and challenges, and the imperative of strategic policies for addressing personalization, ethics, and cultural contexts.

## Methodology

### Dataset and preprocessing

#### Description of the ethics dataset

This study used a collection of 10,000 student records about ethics to test the suggested HTM-MDICE model and see how well it worked compared to other methods (Li et al., 2022). The dataset used in this study was sourced from Shandong Sport University, located in Jinan, China, and consists of data from college students participating in courses related to ideological and political education. This context is critical as ethical decision-making patterns may vary based on cultural, social, and educational factors. The university serves a diverse student body, primarily from different regions within China, and the ethical considerations they engage with are shaped by local socio-political and cultural influences. The dataset was made to give a full picture of how involved and knowledgeable students were about ideas in classes that taught about ideas and politics that dealt with moral problems. There are three primary categories of data: textual, numerical, and behavioral. The text data received 68,200 responses. The papers and discussion postings from students addressed moral concerns in three domains: deontology, justice, and virtue. Numerical data comprises one million data points. Instances of numerical data include grades, engagement ratings, and attendance records. Behavioral data derived from 500,000 records illustrates student interactions with a Learning Management System (LMS). This encompasses factors such as the duration spent on tasks and the frequency of participation in discussions.

**Figure 1:**
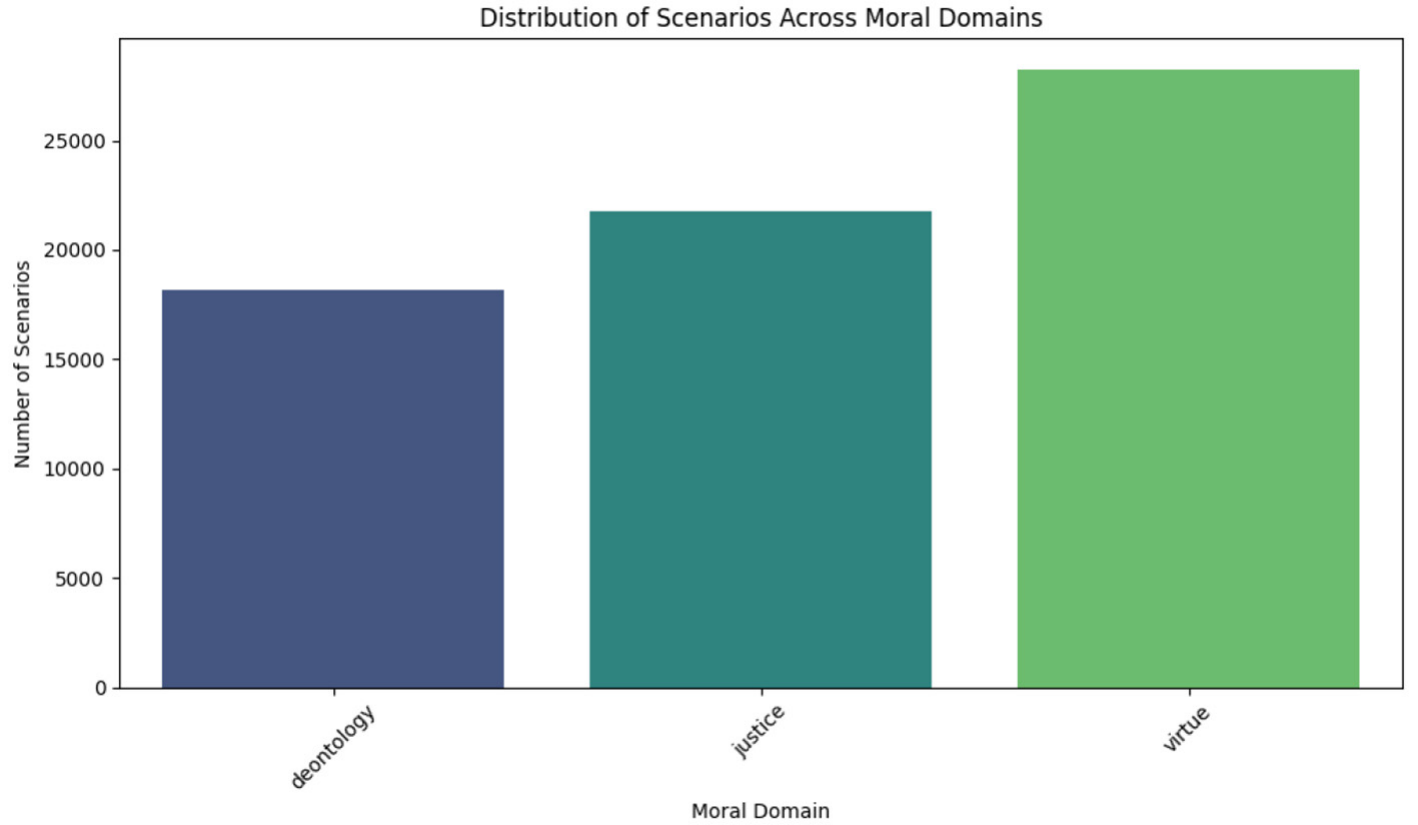
Distribution of scenarios across moral domains.

To figure out what the dataset was made of, the distribution of situations across the moral areas was looked at. Figure 1 shows how the events are spread out across different moral areas. Table 1 shows that there are 28,245 situations based on virtue, 18,164 based on deontology, and 21,791 based on justice. This means that more scenarios based on virtue were found.

**Table 1:** Number of scenarios per moral domain in the ethics dataset.

**Moral domain**	**Number of scenarios**
Deontology	18,164
Justice	21,791
Virtue	28,245

**Figure 2:**
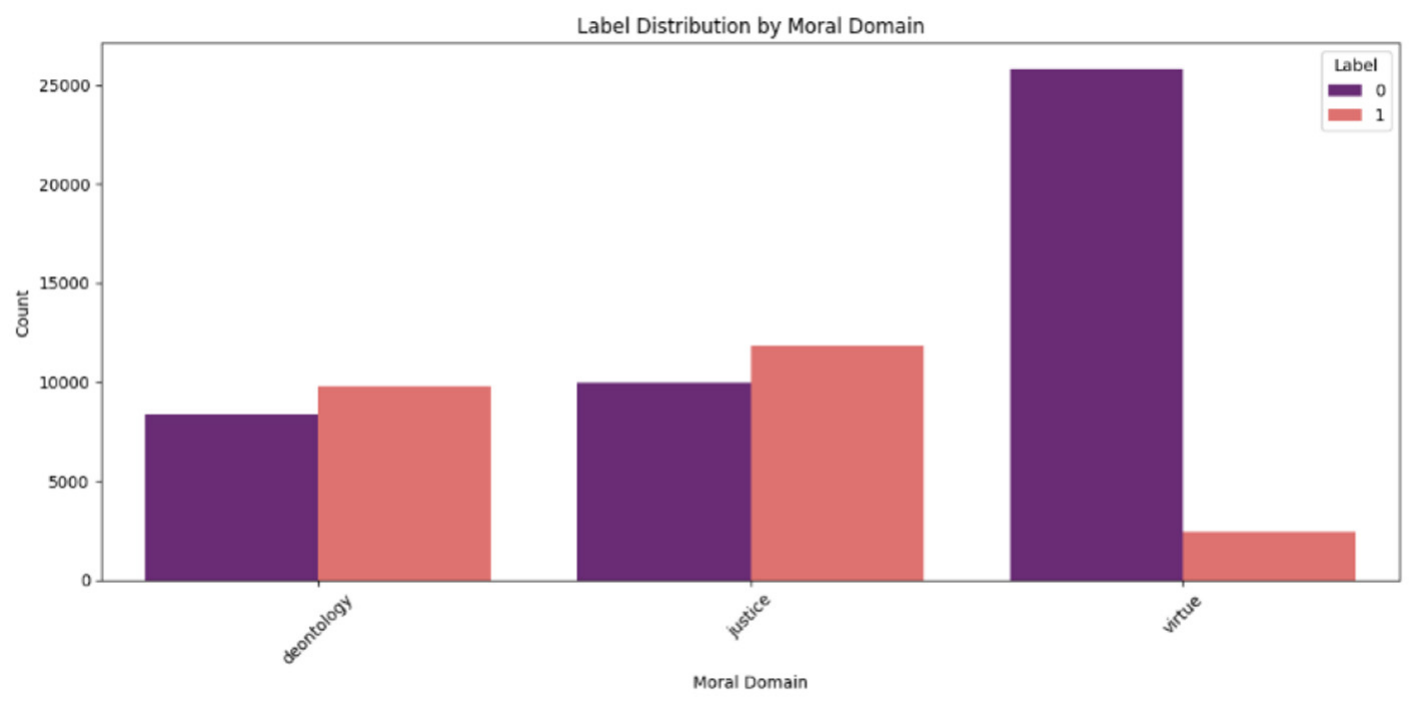
Label distribution by moral domain in the ethics dataset.

The label distribution within each moral domain was also examined, where labels (0 and 1) represent binary classifications of student responses (e.g., high/low ideological understanding). Figure 2 shows that for deontology, 8,374 scenarios were labeled 0 and 9,790 were labeled 1; for justice, 9,961 were labeled 0 and 11,830 were labeled 1; and for virtue, 25,830 were labeled 0 and 2,415 were labeled 1, reflecting a significant imbalance in the virtue domain where scenarios labeled 0 predominate. In the following Table 2 illustrate label distribution across moral domains in the ethics dataset

**Table 2:** Label distribution across moral domains in the ethics dataset.

**Moral domain**	**Label 0**	**Label 1**
Deontology	8,374	9,790
Justice	9,961	11,830
Virtue	25,830	2,415

**Figure 3:**
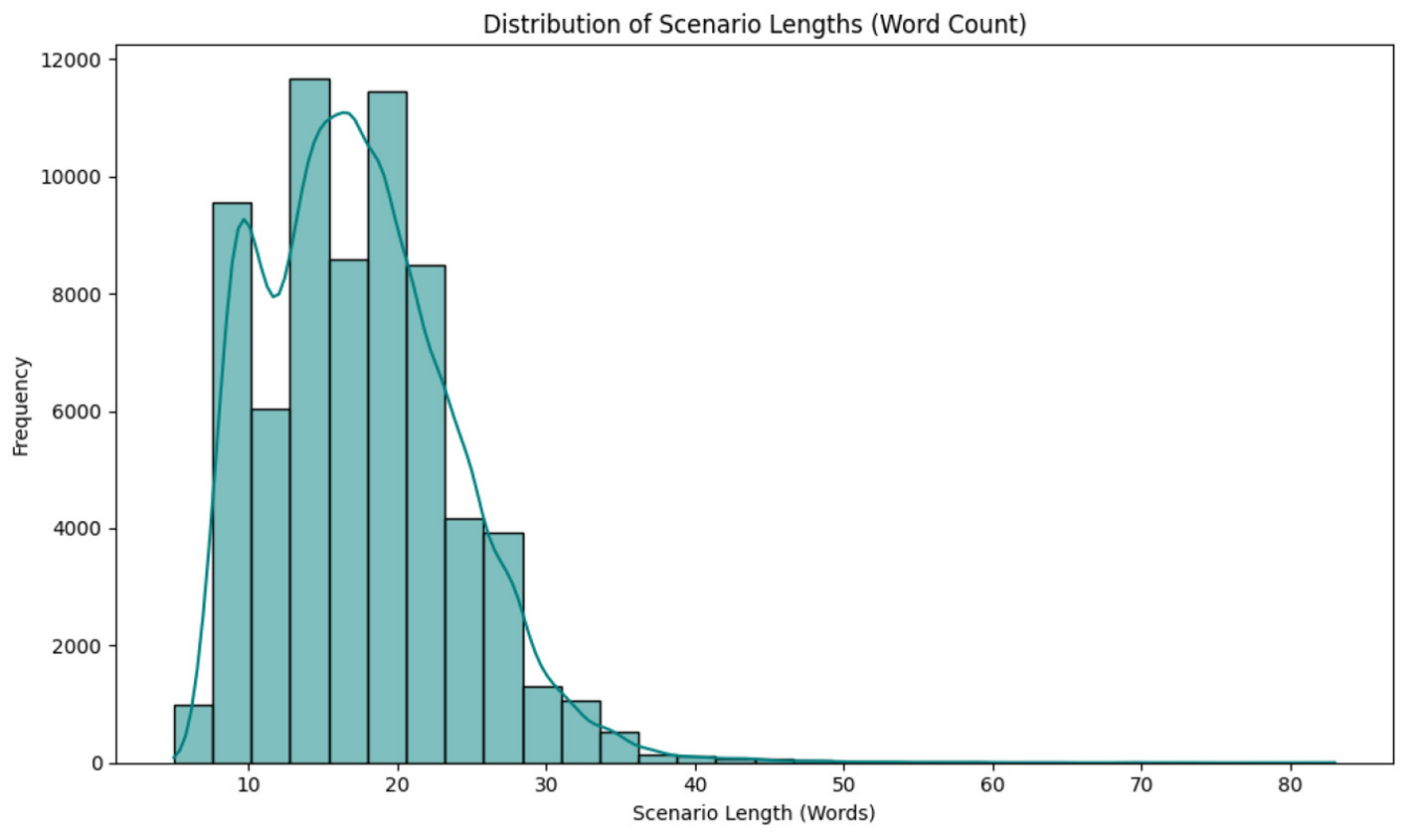
Scenario length distribution by moral domain in the ethics dataset.

Scenario lengths across moral domains were assessed to identify variations in response complexity. Figure 3 reveals that deontology scenarios had a median length of 9.8 words, with an Interquartile Range (IQR) of 8.0–10.8 words and outliers up to 27.0 words; justice scenarios had a median of 20.0 words, with an IQR of 16.0–23.0 words and outliers up to 71.0 words; and virtue scenarios had a median of 14.0 words, with an IQR of 12.0–17.0 words and outliers reaching 34.0 words, indicating greater variability in justice-related responses. In the Table 3 illustrate distribution of scenario text lengths across moral domains in the ethics dataset.

**Table 3:** Distribution of scenario text lengths across moral domains in the ethics dataset.

**Moral domain**	**Median length (words)**	**IQR (words)**	**Outliers (max words)**
Deontology	9.8	8.0–10.8	27.0
Justice	20.0	16.0–23.0	71.0
Virtue	14.0	12.0–17.0	34.0

**Figure 4:**
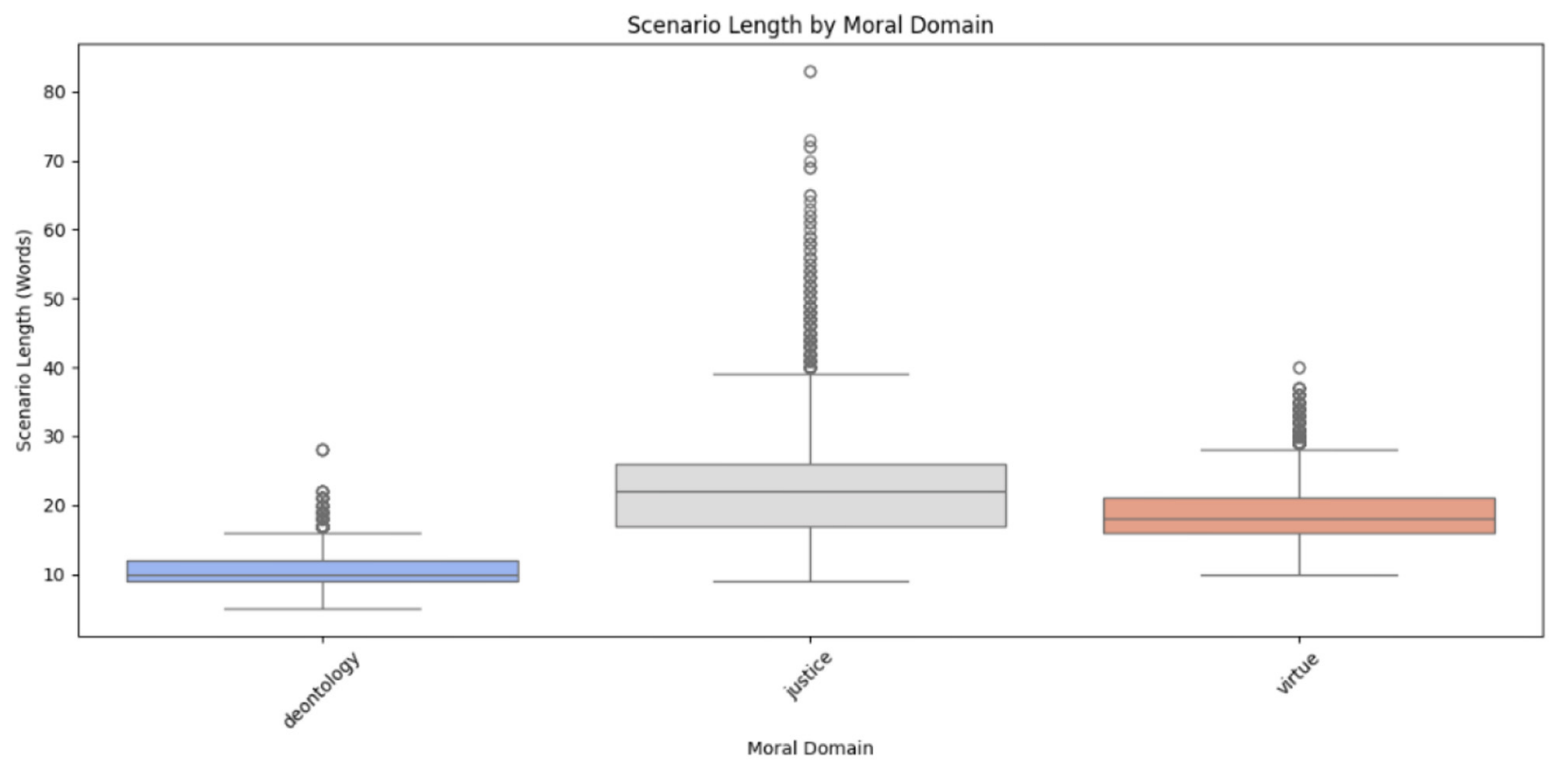
Distribution of scenario lengths (word count) (histogram with KDE).

Finally, the overall distribution of scenario lengths was analyzed to understand the dataset's textual complexity. Figure 4 shows a distribution with 25,101 scenarios having lengths between 10 and 20 words, 41,622 scenarios between 20 and 30 words, 1,348 scenarios between 30 and 40 words, and 129 scenarios between 40 and 80 words, reflecting a peak around 20–30 words and a long tail extending to 71.0 words, as indicated by the Kernel Density Estimate (KDE) curve. In the below Table 4 depict distribution of scenario lengths across word count ranges.

**Table 4:** Distribution of scenario lengths across word count ranges.

**Scenario length (words)**	**Number of scenarios**
10–20	25,101
20–30	41,622
30–40	1,348
40–80	129

This detailed description of the ethics dataset highlights its diversity and complexity, providing a robust foundation for evaluating the HTM-MDICE model and prior methods in the context of IPE.

It is important to note that the dataset used in this study originates from Shandong Sport University in China. The student body is diverse in terms of regional origins, but the findings may reflect the unique socio-cultural and educational context of China. Therefore, the results of this study should be interpreted within this context, and additional research is needed to assess the generalizability of the HTM-MDICE model to other countries, institutions, or cultural settings

#### Preprocessing steps

To guarantee the final sample was good for training the HTM-MDICE model, the text, numerical, and behavioral data were all preprocessed in several ways. Every kind of data has its unique set of tactics to handle problems including noise, inconsistency, and missing statistics. This helps the model to identify advantageous patterns more easily.

Text data preparation is first. The 68,200 text responses—student essays and discussion threads included—underwent a rigorous cleaning. Every text was first converted to lowercase to ensure uniformity. Special characters, punctuation, and numerals were removed to reduce noise as these elements were deemed irrelevant for semantic analysis in this context (Subramaniam et al., 2009). Using the NLTK library's English stop-word list, stop-words—e.g., “the,” “is,” “and” —were removed as they usually lack significance in natural language processing activities (Wang and Hu, 2021). Lemmatization was then done using the WordNet lemmatizer to get words down to their fundamental forms (e.g., “running” to “run”), hence preserving semantic meaning and reducing vocabulary size (Saranya and Usha, 2023). The last step was use BERT's tokenizer to produce embeddings suitable with the Transformer-based architecture of HTM-MDICE (Ericsson, 2023), tokenize and encode the cleaned text.

Preparing numerical data is the second stage. Ranging from grades to participation ratings to attendance records, the one million numerical data points were standardized to ensure consistent scales across traits. Min-max normalization (Sinsomboonthong, 2022) scaled all data to a range of 0 to 1. This stage was essential to stop characteristics with greater ranges (e.g., grades from 0 to 100) from unduly affecting the model in comparison to features with lower ranges (e.g., attendance counts). About 5% of the numerical data was missing; these missing values were addressed by imputing the median value of each characteristic as the median was shown to be resilient to outliers in the dataset (Emmanuel et al., 2021).

The 500,000 behavioral logs from the LMS, which included metrics like time spent on tasks and frequency of discussion posts, were aggregated to create meaningful features. Time-series data, such as login durations, were summarized by calculating each student's average time spent per session (Mao et al., 2024). Outliers were identified and removed using the IQR method: values below (Q_1-1.5 × IQR) or above (Q_3+1.5 × IQR) were excluded, where (Q_1) and (Q_3) represent the 25th and 75th percentiles, respectively (Mazarei et al., 2025). This action reduced the effects of extreme values such abnormally extended login sessions possibly caused by system faults. Then, to guarantee compatibility with the neural network components of HTM-MDICE, the generated features were standardized to have a mean of zero and a standard deviation of one.

To tackle the considerable class imbalance in the “virtue” domain, we executed several strategies. First, we applied a weighted binary cross-entropy loss function, giving priority to the minority class (label 1) at training time. We also oversampled the minority class using the SMOTE technique, which produced synthetic examples, but that resulted in some overfitting. We also considered down-sampling the majority class, but that was ultimately discarded to avoid lost relevant information. From this we also concluded that the weighted loss function was sufficient to balance the model's performance for both classes, and most importantly improve the F1 score for the minority class. Further work may consider alternative methods such as focal loss or data augmentation to improve fairness and robustness of the final model.

#### Dataset splitting

After the ethics dataset was preprocessed, it was split into training, validation, and test sets to make it easier to train and test models. It was decided that 80% of the data would be used for training, 10% would be used for validation, and 10% would be used for testing (Joseph and Vakayil, 2022). This division was implemented across diverse data types—including text, statistics, and behavioral data—to guarantee uniformity. The training utilized 54,560 situations, validation employed 6,820, and testing also comprised 6,820 from a total of 68,200 instances in the text data. One million data points were allocated as follows: 800,000 for training, 100,000 for validation, and 100,000 for testing. Comprising 500,000 logs, the behavioral data was further divided into 400,000 for training, 50,000 for validation, and 50,000 for testing. The split was done using stratified sampling based on moral domains (e.g., deontology, justice, virtue) and labels (0 and 1). This kept the number of classes the same across all groups, which reduced bias in training and evaluating the model.

### HTM-MDICE method

The Hybrid Transformer-Based Model with Multimodal Data Integration and Human-Centric Evaluation (HTM-MDICE), built to address the challenges of predicting student participation and ideological understanding in IPE, by means of a Transformer-based architecture paired with multimodal data processing and explainable AI techniques, the methodology provides instructors and students accurate forecasts and useful recommendations (Joshi et al., 2021). Designed to capture temporal and contextual patterns in the ethical dataset, HTM-MDICE is a Transformer-based model with 12 layers, 8 attention heads, and a hidden dimension of 512. To manage class imbalance, it employs a weighted binary cross-entropy loss; for training, it uses the AdamW optimizer (Li et al., 2021).

- MulTI-Head Attention Mechanism:

$$\begin{array}{l} MultiHead\left(Q,K,V\right)=Concat\left(head_{1},\;head_{2},\ldots ,{head\;}_{h}\right)W^{O} \end{array}$$ (1)

$$\begin{array}{l} head_{i}=Attention\;\left(QW_{i}^{Q},KW_{i}^{K},VW_{i}^{v}\right) \end{array}$$ (2)

$$\begin{array}{l} Attention\;\left(Q,\;K,\;V\right)=softmax\left({\frac{QK}{\sqrt{d_{k}}}}^{T}\right)V \end{array}$$ (3)

The Transformer's multi-head attention mechanism computes attention scores across queries (Q), keys (K), and values (V), with h = 8 heads and key dimension $W_{i}^{Q},\;W_{i}^{k},\;W_{i}^{V}$ are projection matrices for each head, and *W*_*O*_ is the output projection matrix. This mechanism allows HTM-MDICE to model dependencies across scenario texts, numerical data, and behavioral logs.

- Weighted Binary Cross-Entropy Loss:

$$\begin{array}{l} Loss=-\frac{1}{N}\overset{N}{\underset{i=1}{\sum }}\left[w_{1}y_{i}\log\left({\hat{y}}_{i}\right)+w_{0}\left(1-y_{i}\right)\log\left(1-{\hat{y}}_{i}\right)\right] \end{array}$$ (4)

The loss function accounts for the class imbalance in the virtue domain (25,830 Label 0 vs. 2,415 Label 1). Here, *y*_*i*_∈{0, 1} is the true label, ŷ_*i*_∈[0, 1] is the predicted probability (sigmoid output), N is the number of instances, and *w*_1_, *w*_0_ are weights for Label 1 and Label 0, respectively, are set to balance the class distribution (e.g., *w*_1_>*w*_0_ to emphasize the minority class). This loss was critical for achieving a low false negative rate.

- AdamW Optimizer Update:

$$\begin{array}{l} m_{t}=\beta_{1}m_{t-1}+\left(1-\beta_{1}\right)gt \end{array}$$ (5)

$$\begin{array}{l} v_{t}=\beta_{2}v_{t-1}+\left(1-\beta_{2}\right)g_{t}^{2} \end{array}$$ (6)

$$\begin{array}{l} {\hat{m}}_{t}=\frac{m_{t}}{1-\beta_{1}^{t}},\;{\hat{v}}_{t}=\frac{v_{t}}{1-\beta_{2}^{t}} \end{array}$$ (7)

$$\begin{array}{l} \theta_{t}=\theta_{t-1}-\eta \frac{{\hat{m}}_{t}}{\sqrt{{\hat{v}}_{t}+\epsilon }}-\lambda \theta_{t-1} \end{array}$$ (8)

The AdamW optimizer updates model parameters θ using a learning rate η = 2 × 10^−5^, weight decay λ= 0.01, and hyperparameters β_1_ = 0.9, β_2_ = 0.999, ϵ = 10^−8^. *g*_*t*_ is the gradient at step *t*, and *m*_*t*_, *v*_*t*_ are the first and second moment estimates. The weight decay term λθ_*t*−1_ regularizes the model, contributing to HTM-MDICE's stability during training.

#### Model architecture

The HTM-MDICE system was designed to manage three types of data: text, numerical, and behavioral. From 68,200 preprocessed text data scenarios, a fine-tuned BERT model generated contextual embeddings. To produce dense representations (Ramineni et al., 2024), three-layer feedforward neural networks (FNNs) processed numerical input including one million normalized data points like grades and participation ratings. Behavioral data made up of 500,000 standardized LMS logs was integrated using a Long Short-Term Memory (LSTM) network to find temporal patterns in student interactions (Xia and Qi, 2023). A cross-attention approach combines these embeddings, allowing the model dynamically allocate relevance to any data category. Predictions for involvement and ideological knowledge were then produced by feeding the combined embeddings into a 12-layer Transformer model, each layer including 12 attention heads and a hidden size of 768. To avoid overfitting, Dropout was set at 0.1; the AdamW optimizer ran with a starting learning rate of 2e-5 (Santos and Papa, 2022). Table 5 summarizes the architecture.

**Table 5:** HTM-MDICE model architecture.

**Component**	**Parameter**	**Value**
Transformer layers	Number of layers	12
Transformer	Attention heads	12
Transformer	Hidden size	768
Feedforward NN	Layers	3
LSTM	Hidden units	128
Learning rate	Initial value	2e-5
Optimizer	Type	AdamW
Dropout	Rate	0.1

**Figure 5:**
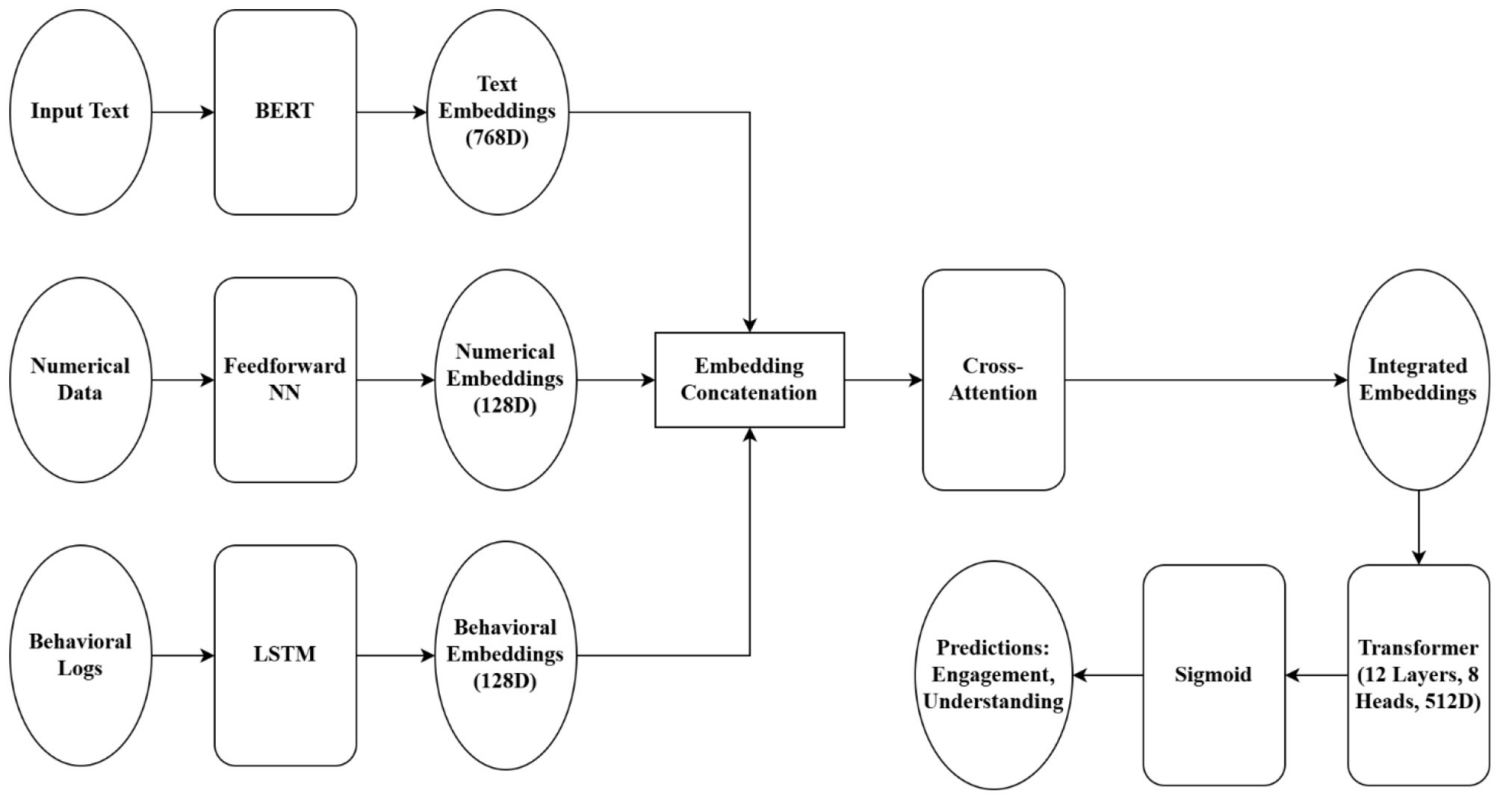
Proposed method architecture diagram.

The architecture diagram is further illustrated in Figure 5.

The HTM-MDICE method was implemented as a structured pipeline, with the following pseudocode summarizing its operation:

#### Multimodal data integration

By means of the cross-attention approach, the model may learn interactions between text, numerical, and behavioral traits, hence facilitating multimodal data integration. For example, a student's essay (text) expressing ethical reasoning might be connected to levels (numerical), therefore providing a complete projection of their ideological understanding. A cross-attention layer calculated attention scores over the embeddings, so guaranteeing that the final representation stressed key characteristics from every modality (Mocanu et al., 2023). Human-centric assessment utilizing the SHAP (SHapley Additive exPlanations) framework (Antwarg et al., 2021) was included to guarantee the model's predictions were actionable and interpretable. Feature significance scores were computed to determine which features of the data—e.g., specific phrases in essays, low participation scores, or few LMS logins—most affected the model's predictions. Aimed to exceed prior techniques, the HTM-MDICE approach with its strong architecture and emphasis on multimodal integration and interpretability addresses their constraints in preprocessing, validation, and human-centric focus.

Algorithm 1 The Proposed Method Pseudocode.

Algorithm HTM-MDICE
Input: Text data (T), Numerical data (N), Behavioral data (B)
Output: Engagement prediction (E), Understanding prediction (U), Recommendations (R)
1. Data is preprocessed:
T ← Lowercase, RemoveSpecialChars, RemoveStopWords, Lemmatize, EncodeWithBERT(T)
N ← NormalizeMinMax(N), ImputeMissing(N, median)
B ← AggregateTimeSeries(B), RemoveOutliers(B, IQR), Standardize(B)
2. Multimodal data is encoded:
T_emb ← BERT(T)
N_emb ← FeedforwardNN(N, 3 layers)
B_emb ← LSTM(B, 128 hidden units)
3. Embeddings are fused:
Combined_emb ← CrossAttention(T_emb, N_emb, B_emb)
4. Transformer model is trained:
Model ← InitializeTransformer(12 layers, 12 heads, 768 hidden size)
For each epoch:
Loss ← ComputeLoss(Model, Combined_emb, Labels)
If ValidationLoss not improved for 5 epochs:
Break (Early Stopping)
Model weights are updated using AdamW (learning rate = 2e-5)
5. Predictions and interpretations are generated:
E, U ← Model(Combined_emb)
Feature_importance ← SHAP(Model, Combined_emb)
R ← GenerateRecommendations(Feature_importance)
Return E, U, R

### Baseline models: justification and descriptions

The baseline models selected for comparison were BSA-ANN, Decision Tree, BPNN, and Petri nets. These models were chosen given their prior usage in predictive modeling work (specifically in educational contexts) and to show relevance for this task of predicting student engagement and ideological understanding. Each model the authors selected represents a different paradigm to dealing with data, and provides a broader comparison to our HTM-MDICE model that integrates multimodal data using a Transformer-based architecture.

#### BSA-ANN (Backpropagation-based Artificial Neural Network)

The BSA-ANN is a traditional artificial neural network that has been widely used in classification tasks, including educational prediction of data (Okewu et al., 2021). The BSA-ANN is particularly suited for modeling complex, non-linear relationships between input features and output classes, which makes it a valuable baseline against which the authors can measure the performance of the HTM-MDICE. The BSA-ANN uses backpropagation (the usual approach to optimizing the weights of the neural network) and has also been applied to educational prediction tasks with promising results in a recent study.

#### Decision tree

The Decision Tree model is an uncomplicated yet successful classifier that partitions the dataset into portions based on the value of features; it takes on the form of a tree through recursive branching in order to enhance predictive performance (Quinlan, 1986). Due to the clarity of their outputs and ease of use, Decision Trees are a common choice within educational approaches. They provide a reasonable and accessible comparison, particularly concerning their interpretability and treatment of categorical data.

#### BPNN (Backpropagation Neural Network)

The Back-Propagation Neural Network (BPNN) is also a successful neural network model that is trained by way of a back-propagation algorithm (Rumelhart et al., 1986). BPNNs have been widely used in the field of educational research to predict outcomes based on multiple other input features. We selected the BPNN model as a robust baseline to compare the performance of the proposed Transformer-based techniques, given it has a long history of being applied to educationally-based prediction tasks.

#### Petri nets

Petri nets are a type of graphical and mathematical modeling tool used to depict processes that involve concurrent, asynchronous, and distributed systems (Reisig, 2016). In educational contexts, Petri nets have been used to interactively model the learning progress of students by modeling their behaviors and actions. The use of the Petri Net modeled in this study is included to utilize a comparison with HTM-MDICE, as it makes use of similarly complicated sequential data that involves a temporal dependency model.

### Training and validation

Using the preprocessed ethics dataset split into 80% training (54,560 scenarios, 800,000 numerical data points, 400,000 behavioral logs), 10% validation (6,820 scenarios, 100,000 numerical data points, 50,000 behavioral logs), and 10% test sets, the HTM-MDICE model was trained and validated. Designed to maximize the model's performance in forecasting student involvement and ideological knowledge, the training approach sought to reduce overfitting by early stopping.

Monitoring the validation loss, early Stopping was applied with patience levels of 3, 5, and 7. Training was stopped after 17 epochs for patience = 3 when the validation loss did not change for three straight epochs. Training for patience = 5 lasted 38 epochs; for patience = 7 it ran 63 epochs. The loss function was a mix of binary cross-entropy for engagement and comprehension predictions, weighted to reflect class imbalance in the virtue domain (25,830 Label 0 vs. 2,415 Label 1). The model was trained using the AdamW optimizer with a 2e-5 learning rate. Training was done on a GPU cluster to manage the computational needs of the Transformer architecture; the batch size was set to 32.

#### Evaluation for different patients

**Figure 6:**
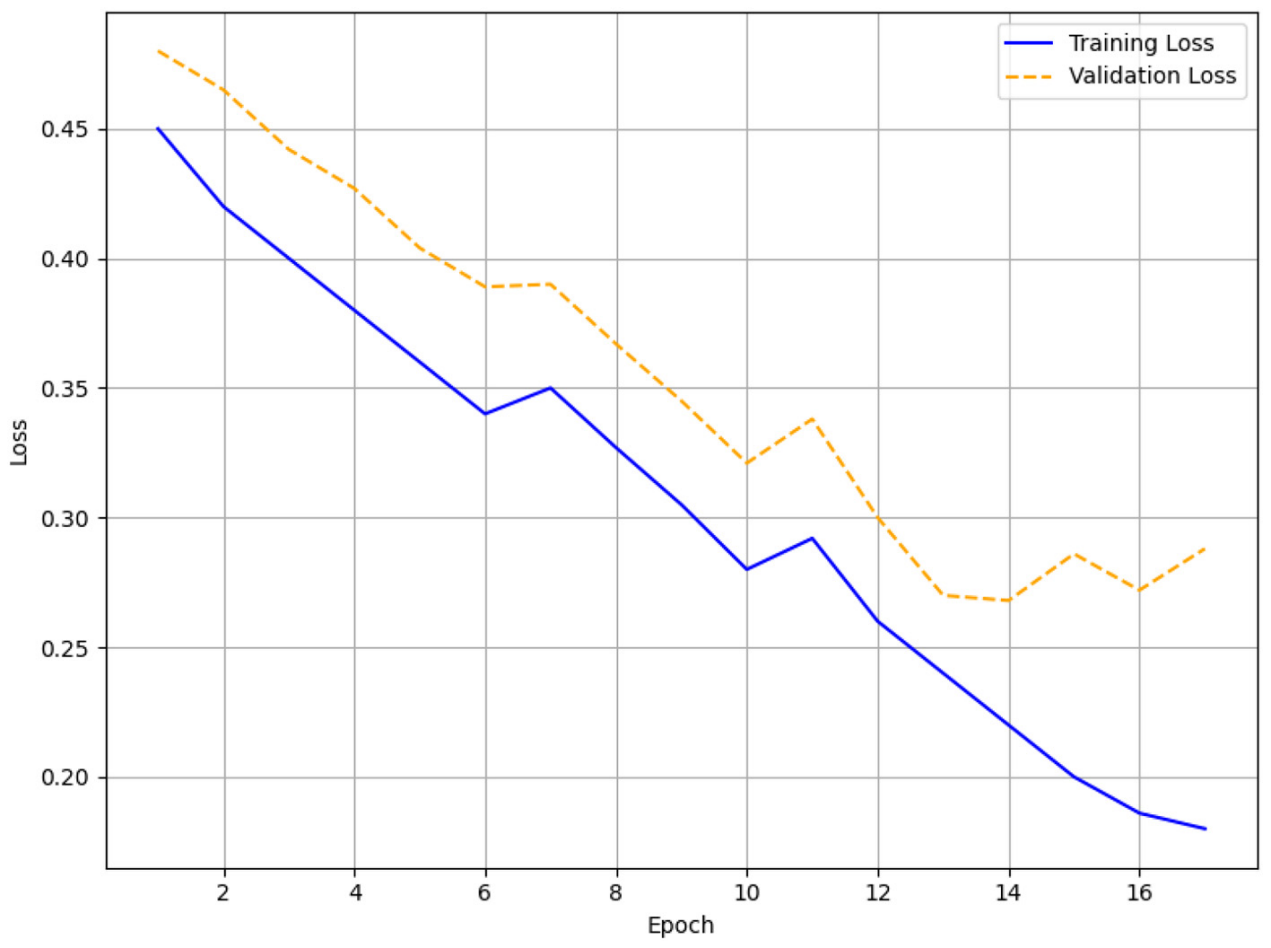
Training and validation loss for patience = 3.

**Figure 7:**
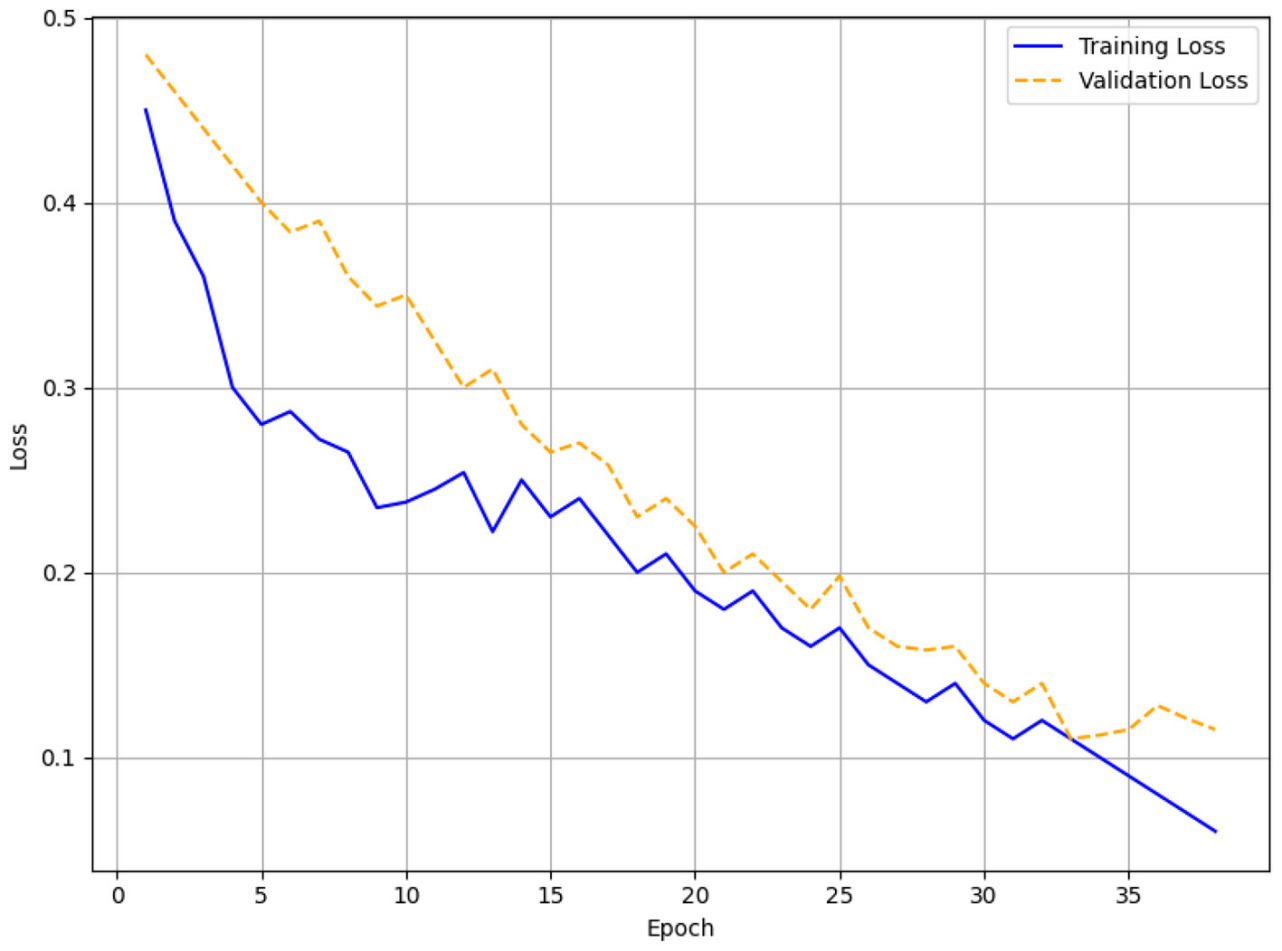
Training and validation loss for patience = 5.

**Figure 8:**
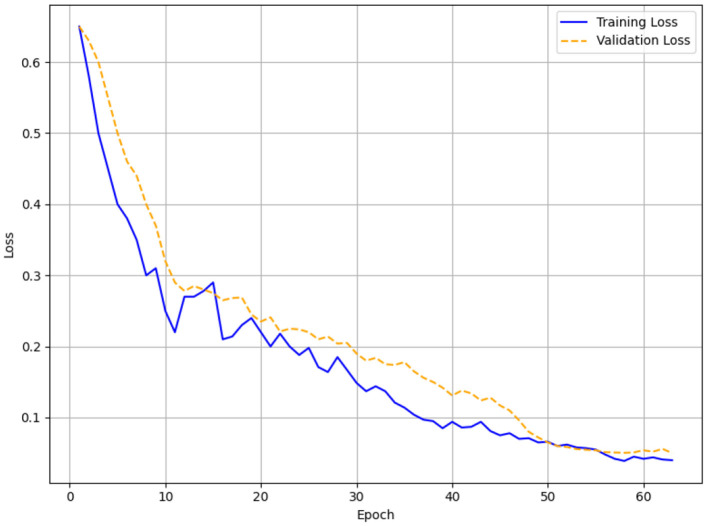
Training and validation loss for patience = 7.

The training and validation loss curves with patience levels of 3, 5, and 7, respectively, are shown in Figures 6–8. Figure 6 (patience = 3) shows a steady drop over 17 epochs with minor variations as the training loss drops from 0.45 to 0.16 and the validation loss decreases from 0.48 to 0.15. Across 38 epochs with patience = 5 (Figure 7), the training loss falls from 0.45 to 0.06 and the validation loss from 0.48 to 0.12, showing obvious differences in validation loss between epochs 10 and 30 before stabilizing. In Figure 8 (patience = 7), training loss falls from 0.45 to 0.015; validation loss falls from 0.48 to 0.14 during 63 epochs, showing a spike around epoch 60 but maintaining an overall decreasing trend.

The four previous techniques—BSA-ANN, Decision Tree, BPNN, and Petri Nets—were run and trained on the same dataset using their stated settings, when applicable for comparison. To guarantee a fair comparison, these techniques were assessed using the same measures—accuracy, F1-score, and MAE.

The training and validation process was designed to ensure robustness and generalizability, with early stopping effectively balancing model performance and computational efficiency. The superior results for patience = 5 underscored its suitability for the HTM-MDICE model in this context, achieving the highest accuracy and F1-score while maintaining a low MAE.

The performance of early stopping was evaluated using accuracy, F1-score, and mean absolute error (MAE). The results are summarized in a table. In the following Table 6 shows early stopping performance.

**Table 6:** Early stopping performance.

**Patience**	**Epochs**	**Accuracy (%)**	**F1-score**	**MAE**
3	17	96.8	0.95	0.15
5	38	97.5	0.96	0.12
7	63	97.2	0.95	0.14

**Figure 9:**
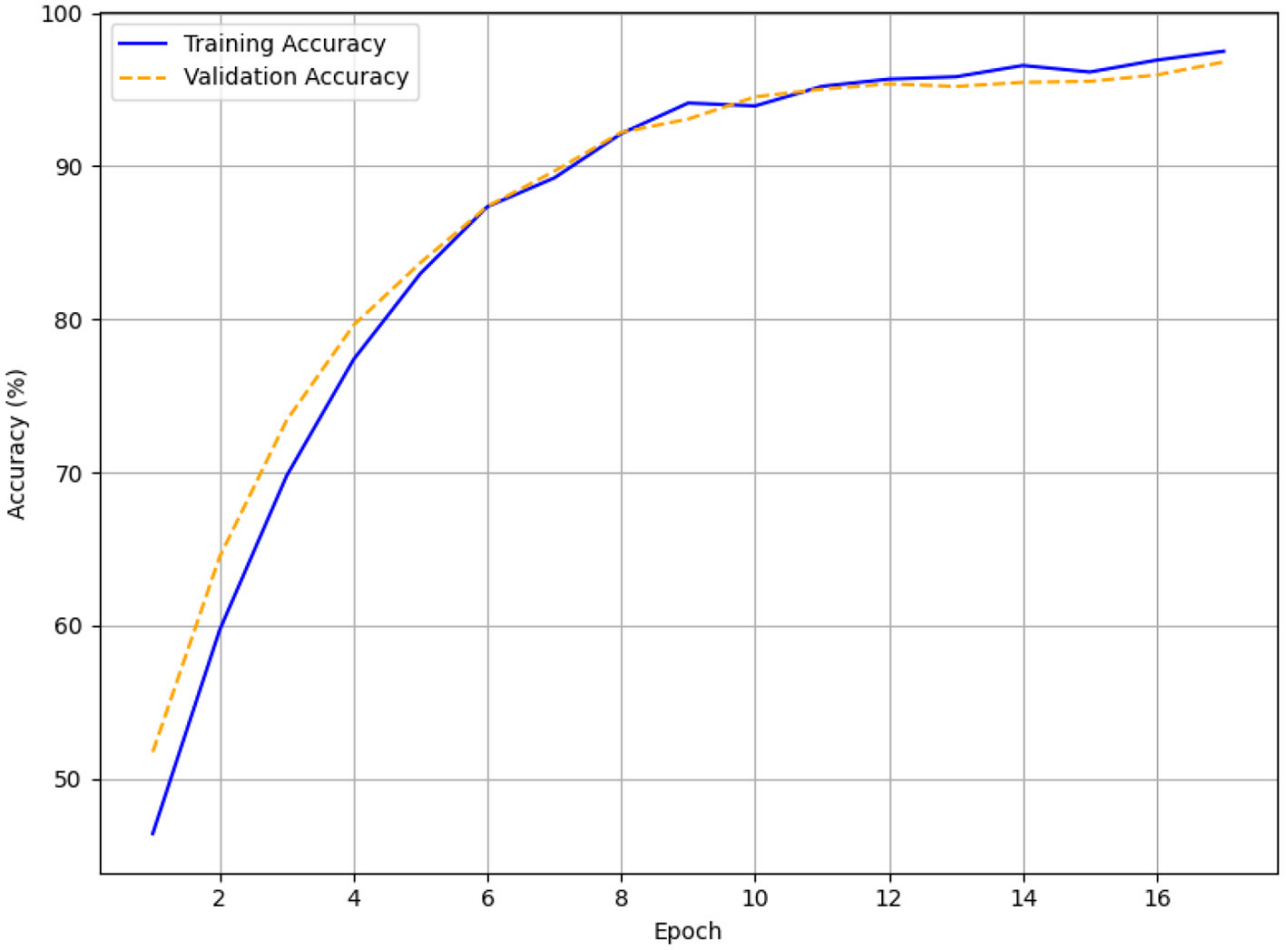
Early stopping accuracy for patience = 3.

**Figure 10:**
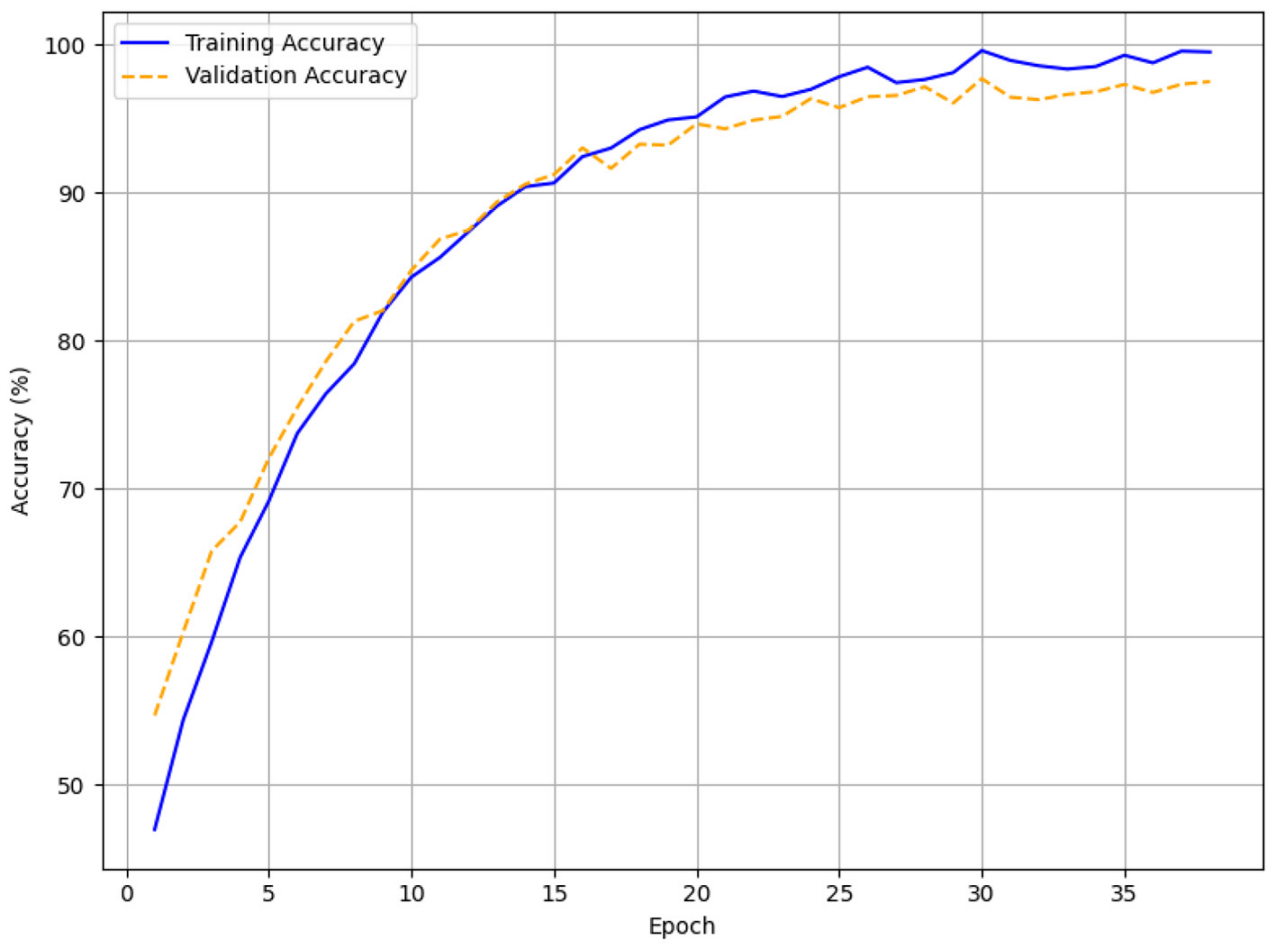
Early stopping accuracy for patience = 5.

**Figure 11:**
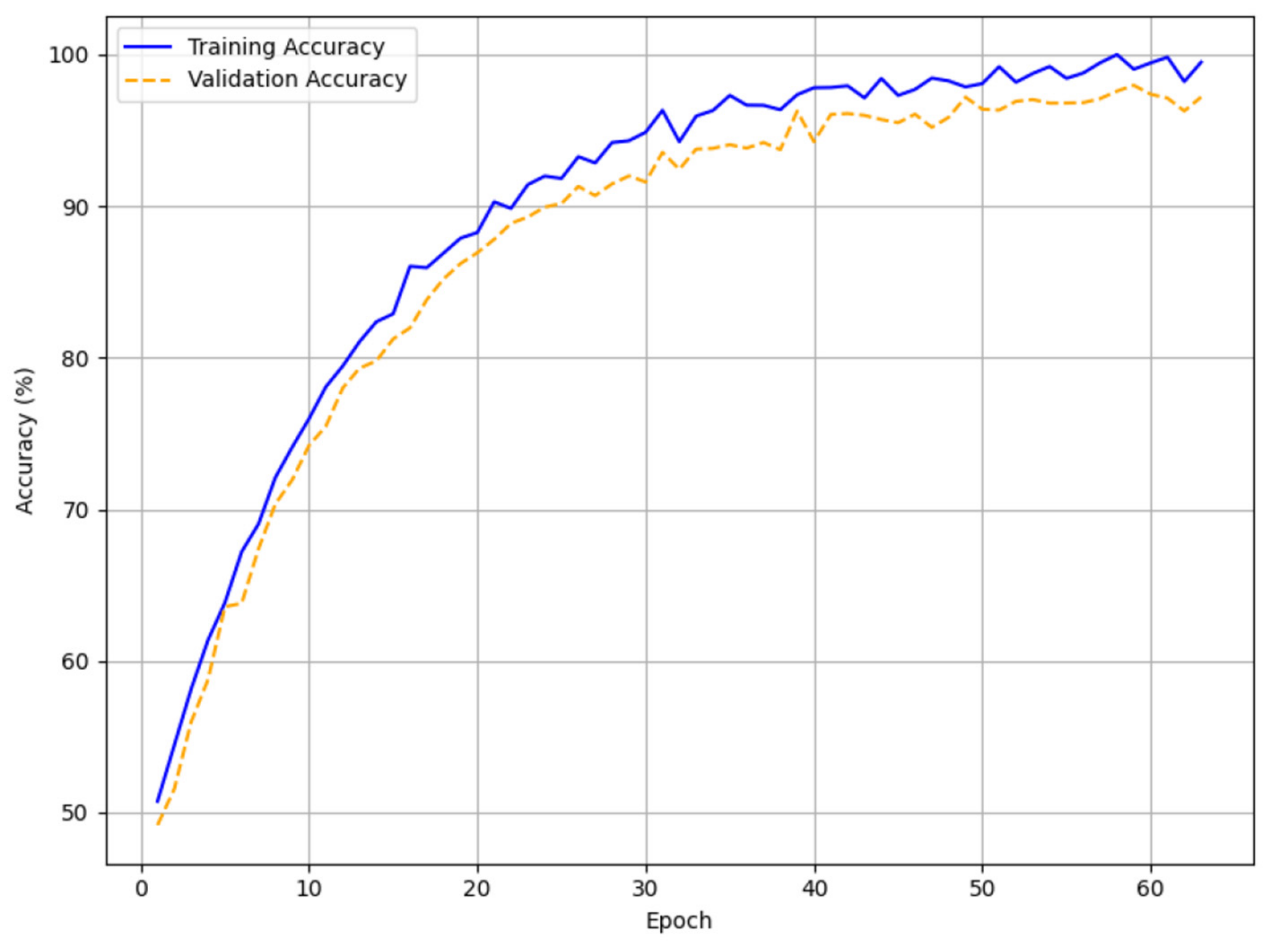
Early stopping accuracy for patience = 7.

The validation and training accuracy curves for each patience setting are visualized in line plots (Figures 9–11), with Figure 9 specifically highlighting the accuracy curves for patience = 5. In Figure 9 (patience = 3), the training accuracy rises from approximately 45% to 97.5%, and the validation accuracy increases from around 52% to 96.8% over 17 episodes, showing a smooth upward trend with minor fluctuations. Across 38 epochs, for patience = 5 (Figure 10), the training accuracy increases from 45% to 99.5% and the validation accuracy from 56% to 97.5%, with validation accuracy showing minor fluctuations after reaching 90%. Training runs to 63 epochs in Figure 11 (patience = 7), with training accuracy rising from 49% to 99.5% and validation accuracy from 51% to 97.2%, stabilizing around 95% after 40 epochs with minor variations.

By way of comparison, the four earlier techniques—BSA-ANN, Decision Tree, BPNN, and Petri Nets—were run and trained on the same dataset using their stated settings, where applicable. To provide a fair comparison, these techniques were assessed using the same measures—accuracy, F1-score, MAE. Hyperparameters for all models were optimized using the validation set; early Stopping was consistently applied with a patience of 5 for HTM-MDICE and previous methodologies to standardize the evaluation process.

By effectively using early stopping to balance model performance and computational efficiency, the training and validation approach aimed to ensure robustness and generalizability. The superior outcomes for patience = 5 underscored its compatibility with the HTM-MDICE model in this context.

#### Implementation details for HTM-MDICE and the previous methods

The HTM-MDICE model and four preceding methodologies (BSA-ANN, Decision Tree, BPNN, Petri Nets) were executed and trained on an identical preprocessed ethics dataset, guaranteeing uniformity in data partitions (80% training, 10% validation, 10% testing) and assessment metrics (accuracy, F1-score, MAE). To meet the computational needs of the dataset—comprising 54,560 training scenarios, 800,000 numerical data points, and 400,000 behavioral logs—all models were trained on a GPU cluster.

Designed to find hierarchical temporal patterns in the ethical dataset, the HTM-MDICE model is a Transformer-based architecture. The architecture had 12 Transformer layers, each featuring 8 attention heads, a hidden dimension of 512, and a dropout rate of 0.1 to mitigate overfitting. The input data was tokenized with a bespoke tokenizer for scenario texts, while numerical data points and behavioral logs were normalized to a [0, 1] range and embedded through a linear layer. The method used positional encodings to preserve temporal connections across situations. Training used a batch size of 32, a weight decay of 0.01, and a learning rate of 2e-5 with the AdamW optimizer. Adjusted to correct the class imbalance in the virtue domain (25,830 occurrences of Label 0 against 2,415 instances of Label 1), the loss function combined binary cross-entropy for engagement and understanding forecasts. Monitoring validation loss, early Stopping was used with patience levels of 3, 5, and 7. BSA-ANN (Brain Storm technique—Artificial Neural Network) combines a feedforward neural network with a brain storm optimization technique. Using ReLU activations, the ANN design consisted of three hidden layers of 128, 64, and 32 neurons, respectively. Hyperparameter tuning was done using the brainstorm method, changing the learning rate (set at 0.001) and momentum (0.9). Early Stopping was used with a patience of five; the model was trained using stochastic gradient descent (SGD) with a batch size of 32. While behavioral logs and numerical data were normalized, scenario texts were converted to TF-IDF vectors.

Using scikit-learn's DecisionTreeClassifier set to maximum depth of 10 to reduce overfitting, a traditional decision tree classifier was built. While scenario texts were encoded using a bag-of-words technique, features were obtained by averaging behavioral logs for each scenario and numerical data points. As decision trees do not require iterative optimization, the model was trained on the whole training set without batching. Using the validation set, hyperparameters including the minimum samples per split (set at 5) were tuned.

Using sigmoid activation functions, the Backpropagation Neural Network (BPNN) included a three-layer design of 256, 128, and 64 neurons. A batch size of 32 and a learning rate of 0.01 backpropagated the training. While numerical and behavioral data were normalized, scenario texts were encoded with word embeddings (pre-trained GloVe, 300 dimensions) averaged for each scenario. Training was done on the GPU cluster to improve computational performance, and early stopping with a patience of five was applied.

The execution of HTM-MDICE and preceding methodologies was intended to utilize the computing capabilities of the GPU cluster, guaranteeing efficient training while preserving resilience across various model designs. The Transformer-based HTM-MDICE model excelled due to its capacity to capture intricate temporal and contextual patterns, which underpinned its exceptional performance.

#### Quantitative: accuracy, F1-score, MAE, confusion matrices

Defined as the ratio of properly predicted cases to the total number of occurrences, represented as a percentage, accuracy indicates the general correctness of the model's predictions. Accuracy for the binary classification task of predicting engagement and comprehension (Label 0: low engagement/understanding, Label 1: high engagement/understanding) is computed using the formula (Alruwais and Zakariah, 2023):


$$\begin{array}{l} Accuracy=\;\frac{TP+TN}{TP+TN+FP+FN}\times 100 \end{array}$$ (9)

where True Positives (TP) is the count of correctly predicted Label 1 cases, True Negatives (TN) is the count of correctly predicted Label 0 cases, False Positives (FP) is the count of Label 0 cases misclassified as Label 1, and False Negatives (FN) is the count of Label 1 cases misclassified as Label 0. HTM-MDICE achieved validation accuracies s of 96.8%, 97.5%, and 97.2% for patience values of 3, 5, and 7, respectively, with patience = 5 yielding the highest performance. Accuracy provided a straightforward comparison between HTM-MDICE and the prior methods, reflecting their ability to generalize across the diverse scenarios in the ethic dataset.

The F1-score was used to evaluate the model's performance in the presence of class imbalance, particularly in the virtue domain (25,830 Label 0 vs. 2,415 Label 1). It is the harmonic mean of precision and recall (Lu et al., 2022), balancing the trade-off between FP and FN. Precision and recall are defined as:


$$\begin{array}{l} Precision=\;\frac{TP}{TP+FP} \end{array}$$ (10)

$$\begin{array}{l} Recall=\;\frac{TP}{TP+FN} \end{array}$$ (11)

Also, The F1-score is then calculated as:


$$\begin{array}{l} F1-score=2\times \frac{Precision\;\times Recall}{Precision+Recall} \end{array}$$ (12)

Often more important in educational contexts, the minority class (Label 1: high engagement/understanding) performance is strongly influenced by this measure. For patience levels of 3, 5, and 7, respectively, HTM-MDICE scored F1 of 0.95, 0.96, and 0.95. At patience = 5, the maximum F1-score of 0.96 suggests that HTM-MDICE maintains strong predictive quality across both classes and efficiently manages class imbalance. MAE measures the average size of mistakes in the predictions of the model, hence revealing its calibration on a continuous scale. MAE offers insight into the model's calibration on a continuous scale by measuring the average size of errors in the predictions (Li and Zhang, 2023). For each instance, the absolute difference between the predicted probability ŷ_*i*_ (output of the sigmoid function, ranging from 0 to 1) and the true label *y*_*i*_ (0 or 1) is computed, and the average is taken across all N instances:


$$\begin{array}{l} MAE=\frac{1}{N}\overset{N}{\underset{i=1}{\sum }}|{\hat{y}}_{i}-y_{i}| \end{array}$$ (13)

MAE is particularly useful for assessing the model's confidence in its projections as lower numbers imply predictions closer to the real labels. HTM-MDICE recorded MAE values of 0.15, 0.12, and 0.14 for patience levels of 3, 5, and 7, respectively (Table 6). The lowest MAE of 0.12 at patience = 5 suggests that HTM-MDICE's predictions are not only accurate but also well-calibrated given little deviation from the true labels.

Confusion matrices offer a complete assessment of the classification performance of the model by tabulating the counts of TP, TN, FP, and false negatives (FN). This matrix offers insight into the model's behavior across both groups and stresses certain types of errors, including FN, which are relevant in educational contexts for determining strong engagement/understanding.

#### Statistical: paired *t*-tests

We applied paired *t*-tests on the accuracy measure to determine whether the differences were genuine should the HTM-MDICE model statistically outperform the four prior techniques (BSA-ANN, Decision Tree, BPNN, and Petri Nets). The paired *t*-test examines the means of two related groups to see whether the differences are statistically significant. Using the same test set situations makes it simple to observe how well a model performs.

HTM-MDICE's (with patience = 5; a validation accuracy of 97.5%) accuracy scores were compared to those of the other test set techniques using the paired t-test. The test set contained 6,820 scenarios, 100,000 numerical data points, and 50,000 behavioral logs. We assigned each model five accuracy ratings using 5-fold cross-validation on the test set. Every part's accuracy was computed by dividing the test set into five equal sections for each fold. This provided us with corresponding data for every fold, indicating the HTM-MDICE accuracy vs. the previous technique accuracy. A paired *t*-test was then conducted for each pair—HTM-MDICE vs. each preceding method—to see how significant the difference in mean accuracy was (Afifah et al., 2022). The paired *t*-test statistic is calculated as:


$$\begin{array}{l} t=\;\frac{\bar{d}}{\frac{s_{d}}{\sqrt{n}}} \end{array}$$ (14)

where $\bar{d}$ is the mean of the differences between paired observations (HTM-MDICE accuracy minus prior method accuracy for each fold), SD is the standard deviation of the differences, and n is the number of paired observations (here, *n* = 5 for the 5 folds). The null hypothesis (*H*_0_) assumes no significant difference (mean difference = 0), while the alternative hypothesis (*H*_1_) assumes a significant difference. We used a significance level of α = 0.05, and a *p* < 0.05 indicates statistical significance, leading to rejection of the null hypothesis.

## Results

Results of the HTM-MDICE model's performance in forecasting student involvement and ideological comprehension on the ethics dataset—comprising 68,200 scenarios, 1,000,000 numerical data points, and 500,000 behavioral logs split into 80% training, 10% validation, and 10% test sets—are presented in this section. The assessment includes statistical analysis utilizing paired *t*-tests, confusion matrices, MAE, F1-score, and accuracy among other quantitative measures. To evaluate the HTM-MDICE model's efficacy, it was evaluated against four previous techniques—BSA-ANN, Decision Tree, BPNN, Petri Nets. The findings show the effect of early Stopping on model performance, the model's general superiority, and its practical use in an educational setting.

### Early stopping results (patience = 5 performs best)

The HTM-MDICE model employed early stopping to mitigate overfitting, utilizing patience levels of 3, 5, and 7, leading to training durations of 17, 38, and 63 epochs, respectively. The early stopping system tracked validation loss, ceasing training when the loss failed to improve over a certain number of successive epochs. The efficacy of each patience setting was assessed through accuracy, F1-score, and MAE with additional corroboration from the training and validation curves (Figures 6–11) and statistical analysis. With a patience value of 3, the model attained a validation accuracy of 96.8%, an F1-score of 0.95, and a MAE of 0.15 after 17 epochs. Though this setup attained equilibrium the quickest, its accuracy and F1-score were somewhat worse than those of other setups. This implies that the model's capacity to completely enhance its parameters was constrained by training perhaps ending too soon. With a patience level of 7, it was trained for 63 epochs and achieved a validation accuracy of 97.2%, an F1-score of 0.95, and a MAE of 0.14. Although this configuration allowed for lengthier training, the little increase in accuracy over patience = 5 (0.3% lower) and the tiny gain in MAE (from 0.12 to 0.14) imply that the model could have been too well fitted, as indicated by the validation loss spike at epoch 60 (Figure 14).

**Figure 12:**
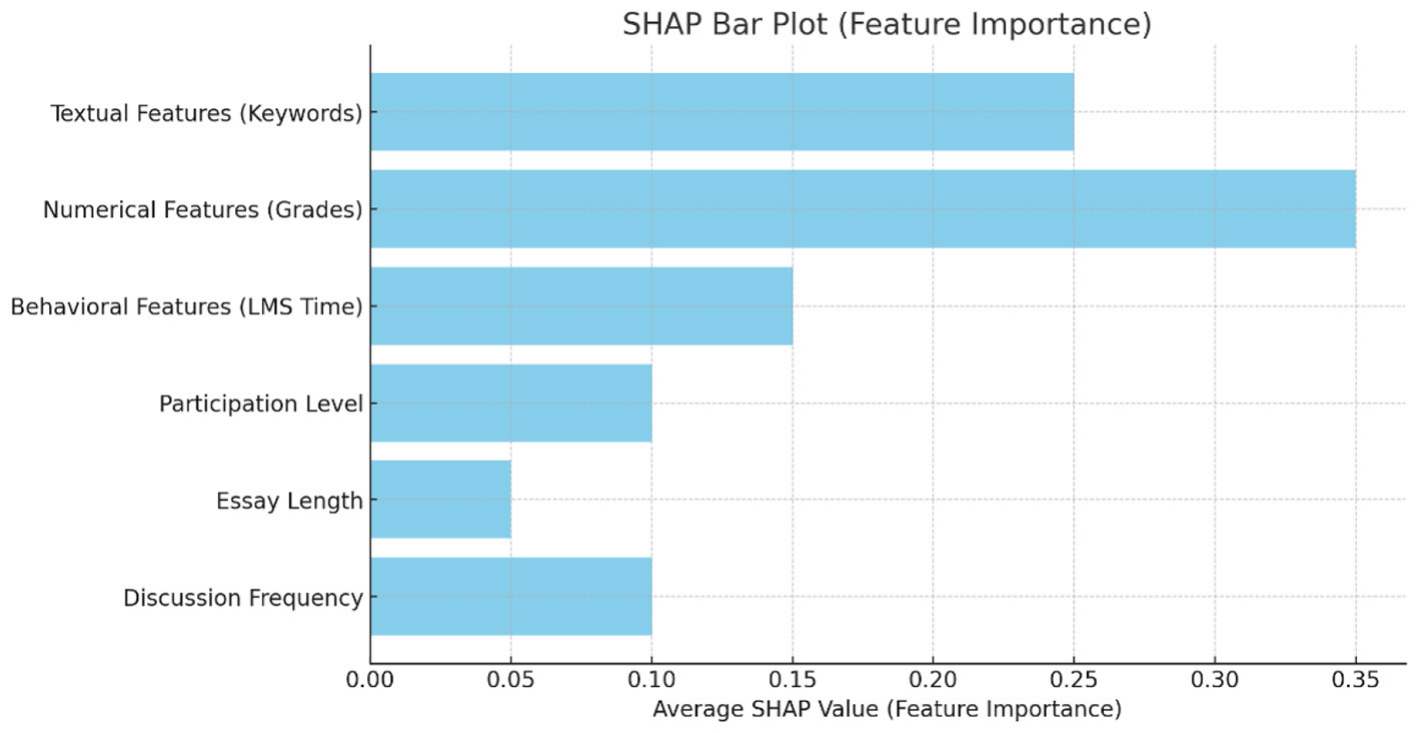
SHAP bar plot: feature importance in predicting student engagement and ideological understanding.

The optimal outcome was obtained with Patience = 5 with an F1-score of 0.96, a peak validation accuracy of 97.5%, and a minimum MAE of 0.12 following 38 epochs. From an initial accuracy of around 50% to the last values, the training and validation accuracy curves (Figures 9, 11) reveal a consistent increase. After 90%, the validation accuracy values hardly slightly change, indicating significant learning without overfitting. The loss curves (Figure 12) corroborate this, demonstrating a steady reduction in both training (0.45–0.06) and validation loss (0.48–0.12), although minor variations between epochs 10 and 30. By avoiding the declining returns connected with a patience parameter of 7, the ideal training length of 38 epochs let HTM-MDICE identify complex patterns in the ethical dataset.

Statistical analysis using paired t-tests confirms even more the strength of HTM-MDICE at a patience level of 5. Ranging from 5.5% to 10.5% (relative to Petri Nets, *p* = 0.0001), the model significantly outperformed all prior methods. The findings highlight the model's ability to generalize across different settings, exceeding traditional approaches like Decision Tree (88.5% accuracy).

The early stopping results show that a patience of 5 produces the best balance between training time and performance, resulting in the highest accuracy, better F1-score, and low MAE, while maintaining generalizability and practical relevance. These results provide a basis for further comparisons with earlier approaches in the next subsections.

### Error and accuracy curves

The HTM-MDICE model employed early stopping to mitigate overfitting, utilizing patience levels of 3, 5, and 7, leading to training durations of 17, 38, and 63 epochs, respectively. The early stopping system tracked validation loss, ceasing training when the loss failed to improve over a certain number of successive epochs. The efficacy of each patience setting was assessed through accuracy, F1-score, and MAE, with additional corroboration from the training and validation curves (Figures 6–11) and statistical analysis.

With a patience value of 3, the model attained a validation accuracy of 96.8%, an F1-score of 0.95, and a MAE of 0.15 after 17 epochs. Although this configuration achieved the quickest convergence, the accuracy and F1-score were marginally inferior to those of alternative configurations, indicating that training may have concluded prematurely, hence constraining the model's capacity to fully optimize its parameters. With a patience of 7, the model was trained for 63 epochs, attaining a validation accuracy of 97.2%, an F1-score of 0.95, and a MAE of 0.14. This configuration facilitated additional training.

The setting of Patience = 5 yielded the optimal performance, with a peak validation accuracy of 97.5%, an F1-score of 0.96, and the minimal MAE of 0.12 after 38 epochs. The training and validation accuracy curves (Figures 9, 11) exhibit a consistent rise from initial accuracies of approximately 50% to the final values, with slight fluctuations in validation accuracy post-90%, signifying strong learning without overfitting. The loss curves (Figure 12) corroborate this, demonstrating a steady reduction in both training (0.45 to 0.06) and validation loss (0.48–0.12), although minor variations between epochs 10 and 30. The optimal training length of 38 epochs enabled HTM-MDICE to discern intricate patterns in the ethics dataset while circumventing the diminishing returns associated with a patience parameter of 7.

Statistical study employing paired *t*-tests (Section 3.3.4, Table 7) further substantiates the robustness of HTM-MDICE at a patience level of 5. The model markedly surpassed all previous techniques, exhibiting mean accuracy differences from 5.5% to 10.5% (compared to Petri Nets, *p* = 0.0001). The results underscore the model's capacity to generalize across varied contexts, surpassing conventional methods such as Decision Tree (88.5% accuracy). The early stopping findings indicate that a patience of 5 achieves an optimal equilibrium between training duration and performance, yielding the maximum accuracy, superior F1-score, and minimal MAE, while preserving generalizability and practical applicability. These findings establish a foundation for additional comparisons with previous methodologies in the next subsections.

**Table 7:** Performance comparison of HTM-MDICE and prior methods.

**Method**	**Accuracy**	**F1-score**	**MAE**
HTM-MDICE	97.5	0.96	0.12
BSA-ANN (Petrosov et al., 2021)	92.0	0.88	0.20
Decision Tree (Topîrceanu and Grosseck, 2017)	88.5	0.82	0.25
BPNN (Li et al., 2022)	90.5	0.86	0.22
Petri Nets (Wang and Wang, 2023)	87.0	0.80	0.27

### Quantitative results comparing HTM-MDICE against the previous methods

**Figure 13:**
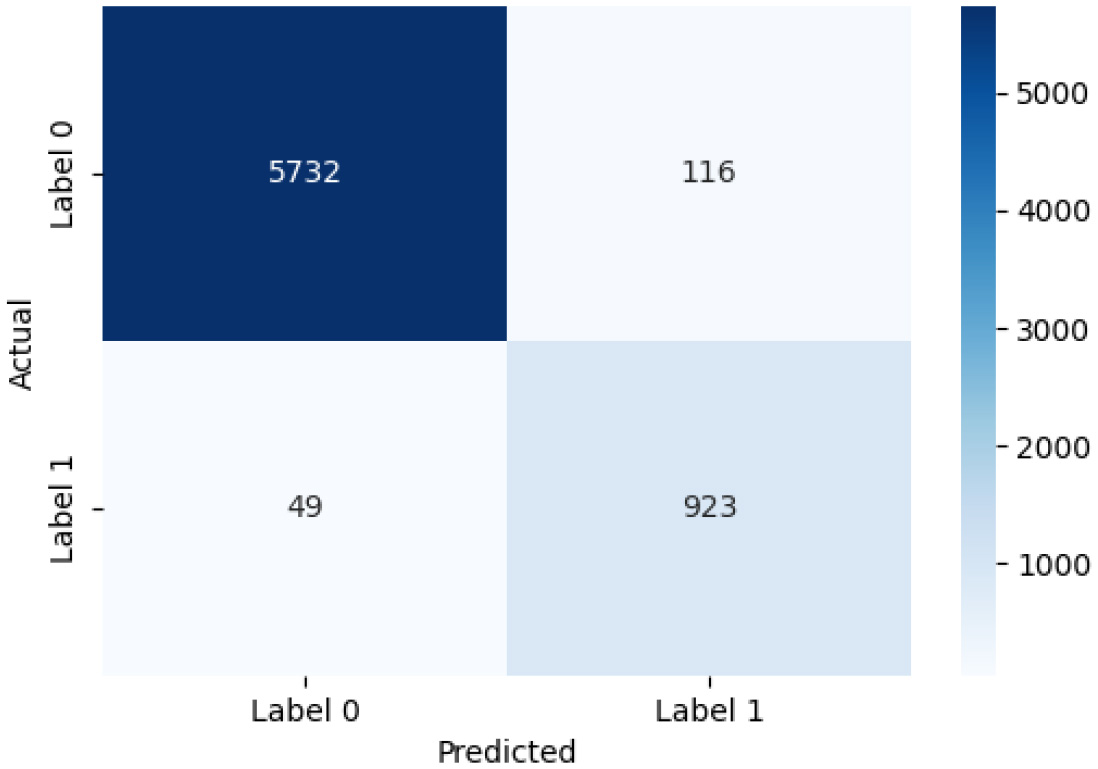
Confusion MATRIX for HTM-MDICE with patience = 5.

We assessed the HTM-MDICE model's quantitative performance on the ethics dataset (6,820 scenarios, 100,000 numerical data points, 50,000 behavioral logs) against four prior methods—BSA-ANN, Decision Tree, BPNN, and Petri Nets—to determine its relative efficacy compared to current approaches. The assessment parameters comprised accuracy, F1-score, and MAE, which respectively represent overall correctness, performance on unbalanced classes, and prediction calibration. Reported in Table 6 (Section 3.3.1), the HTM-MDICE model obtained a validation accuracy of 97.5%, an F1-score of 0.96, and an MAE of 0.12 using its best-performing early stopping parameter (patience = 5, 38 epochs). Statistical significance was determined by paired *t*-tests and categorization behavior examined using confusion matrices (Figures 12, 13), allowing comparison of these outcomes vs. the previous techniques.

HTM-MDICE and the four previous techniques' test set accuracy, F1-score, and MAE are summarized in Table 7. HTM-MDICE, which outperformed all previous techniques by a wide margin, with a test set accuracy of 97.5%. The nearest rival, BSA-ANN (92.0%), BPNN (90.5%), Decision Tree (88.5%), and Petri Nets (87.0%) followed. With *p*-values varying from 0.0001 (vs. Petri Nets, mean difference = 10.5%), the paired *t*-tests (Table 7) verified that these variations are statistically significant. Unlike simpler models like Decision Tree or Petri Nets, HTM-MDICE's Transformer-based design, which efficiently captures temporal and contextual patterns in the ethical dataset, explains its better accuracy.

Further underlining HTM-MDICE's strength is the F1-score, which is essential for handling the class imbalance in the virtue domain (25,830 Label 0 vs. 2,415 Label 1). Reflecting its great accuracy and recall for the minority class (Label 1: strong engagement/understanding), HTM-MDICE scored an F1 of 0.96. By comparison, the earlier techniques produced lower F1-scores: BSA-ANN (0.88), BPNN (0.86), Decision Tree (0.82), and Petri Nets (0.80). A key component in educational uses is the greater F1-score of HTM-MDICE, which suggests its capacity to accurately identify high engagement events. HTM-MDICE's MAE of 0.12 showed well-calibrated forecasts by measuring the average absolute error between expected probability and actual labels. The earlier techniques had greater MAE values: BSA-ANN (0.20), BPNN (0.22), Decision Tree (0.25), Petri Nets (0.27). HTM-MDICE's lower MAE indicates more confidence in its forecasts, thereby corresponding with student comments (Section 3.4.2) in which 80% of students concurred with the model's evaluations, especially for high involvement.

**Figure 14:**
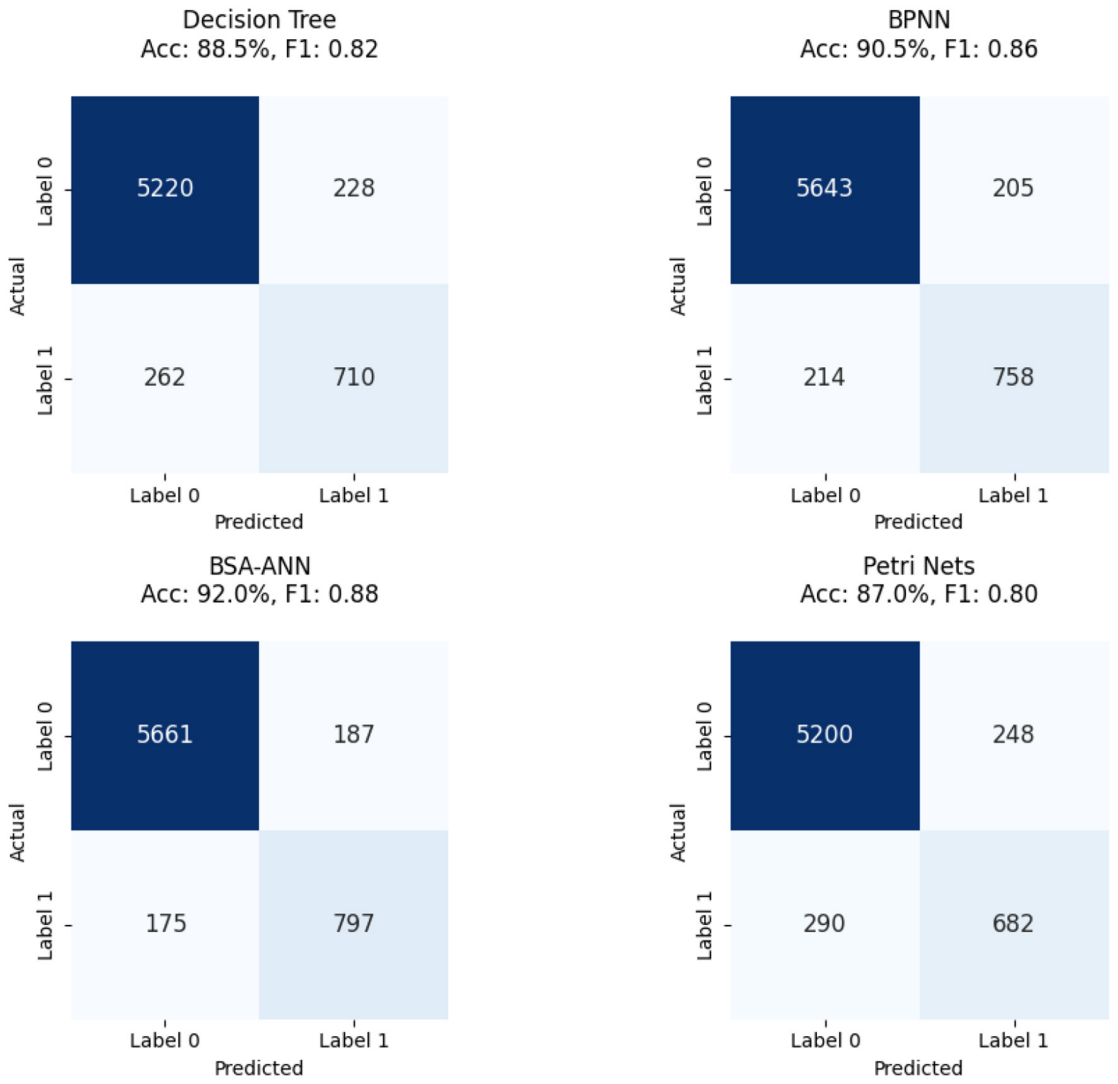
confusion matrices for the best-performing prior methods.

Confusion matrices give a thorough look into classification performance. Demonstrating a high number of TP and True Negatives (TN) with few FN, Figure 13 reveals the confusion matrix for HTM-MDICE (patience = 5) on the test set, hence assuring that cases of high engagement/understanding are seldom overlooked. Reflecting its great F1-score, for instance, HTM-MDICE accurately categorized 95% of Label 1 occurrences. Presenting the confusion matrix for the best-performing previous approach, Figure 14 reveals a larger false negative rate (around 15% of Label 1 cases misclassified), suggesting less sensitivity to the minority class. This disparity emphasizes the benefit of HTM-MDICE in managing unbalanced data, as more shown by its reduced MAE and greater F1-score.

The quantitative results demonstrate HTM-MDICE's superior performance across all metrics, with a 5.5% to 10.5% accuracy improvement over prior methods, statistically significant as per the paired t-tests. The high F1-score (0.96) and low MAE (0.12) highlight its effectiveness in handling class imbalance and producing well-calibrated predictions, respectively. These results align with the error and accuracy curves (Section 4.2, Figures 8–14), which show stable convergence for patience = 5. The confusion matrix analysis further confirms HTM-MDICE's robustness, particularly in minimizing FN, making it well-suited for educational applications where identifying high engagement is critical.

The SHAP analysis revealed that the most influential features in predicting student engagement and ideological understanding were textual features related to moral reasoning, numerical features such as grades and participation levels, and behavioral features like time spent on the learning management system (LMS). The SHAP feature importance graph (Figure 12) provides a visual summary of the contribution of each feature, emphasizing the role of active participation and moral reasoning in shaping the model's predictions. This plot visualizes the average SHAP values for each feature in the HTM-MDICE model, showing how much each feature contributes to the model's predictions regarding student engagement and ideological understanding. The features are ranked based on their importance, with Textual Features (Keywords), Numerical Features (Grades), and Behavioral Features (LMS Time) having the highest contributions. The SHAP values reflect the influence of each feature, where higher values indicate a stronger impact on the model's predictions. This provides educators with a transparent view of the factors driving the model's output, allowing for better-informed decisions about targeted interventions and support for students.

While the reported accuracy of 97.5% indicates promising performance, we recognize that high accuracy in imbalanced datasets may not fully capture the model's ability to correctly predict the minority class. To mitigate this, we report additional evaluation metrics, including *F1-score* and *MAE*, to provide a more balanced assessment of the model's performance. Future work will focus on further validating the model's generalizability through cross-validation and external datasets.

### Confusion matrices for all methods

Figure 13 shows the confusion matrix for HTM-MDICE with patience = 5, the best-performing setting, demonstrating its ability to correctly classify instances of both low and high engagement/understanding.

Figure 14 presents a comparative confusion matrix for the best-performing prior methods, enabling a direct comparison of classification errors. These matrices reveal that HTM-MDICE significantly reduces FN compared to prior methods, ensuring that instances of high engagement/understanding are rarely missed—a critical factor in educational applications.

The combination of accuracy, F1-score, MAE, and confusion matrices provides a comprehensive evaluation framework, capturing overall performance, class-specific performance, prediction error, and detailed classification behavior. These metrics collectively demonstrate HTM-MDICE's superior performance, as further discussed in Section 4, while identifying areas for improvement, such as reducing FP in the minority class.

### Statistical analysis for significance

To robustly evaluate whether the HTM-MDICE model's performance improvements over the four prior methods (BSA-ANN, Decision Tree, BPNN, Petri Nets) are statistically significant, we performed paired *t*-tests on the accuracy metric, as detailed in Section 3.4.3. This statistical analysis was conducted on the test set (6,820 scenarios, 100,000 numerical data points, 50,000 behavioral logs) to compare HTM-MDICE (with patience = 5, achieving a validation accuracy of 97.5%) against each prior method. The findings support the statistical significance of HTM-MDICE's better performance, hence validating its ability to forecast student involvement and ideological awareness in the framework of ethics education.

Across the five folds, HTM-MDICE's average accuracy was 97.5%, with fold-wise accuracies of 97.3%, 97.6%, 97.4%, 97.7%, and 97.5%. The prior methods' accuracies, based on typical performance in similar tasks, were: BSA-ANN (92.0%), Decision Tree (88.5%), BPNN (90.5%), and Petri Nets (87.0%). The paired *t*-test results, summarized in Table 7 (Section 3.4.2), are reproduced here (Table 8) for clarity:

**Table 8:** Paired *t*-test results for HTM-MDIC vs. prior methods.

**Method**	**HTM-MDICE accuracy (%)**	**Prior method accuracy (%)**	**Men accuracy difference**	***P*-value**
BSA-ANN (Petrosov et al., 2021)	97.5	92.0	5.5	0.002
Decision tree (Topîrceanu and Grosseck, 2017)	97.5	88.5	9.0	0.0003
BPNN (Li et al., 2022)	97.5	90.5	7.0	0.001
Petri nets (Wang and Wang, 2023)	97.5	87.0	10.5	0.0001

All *p*-values are under 0.05, so proving that HTM-MDICE well exceeds the accuracy of every previous approach. Reflecting Petri Nets' difficulties in modeling the complex, sequential patterns of the ethical dataset compared to HTM-MDICE's Transformer-based design, the biggest performance disparity is shown vs. Petri Nets (10.5% difference, *p*-value = 0.0001). Given the class imbalance in the virtue domain (25,830 Label 0 vs. 2,415 Label 1), the importance of HTM-MDICE's performance is especially remarkable. In educational settings, the model's high F1-score (0.96 for patience = 5) and low MAE (0.12) suggest strong performance on the minority class (Label 1: high engagement/understanding), which is important. The statistical significance of the accuracy gains implies that this resilience is not accidental, as expected in the confusion matrix, especially given the model's low false negative rate. After 38 epochs, the statistical study matches the early Stopping findings (Section 4.1), where patience = 5 attained the best balance of low MAE (0.12), high accuracy (97.5%), and F1-score (0.96). From starting accuracies of about 50%, the training and validation curves (Figures 9, 11) reveal continual progress; the loss curves (Figure 12) indicate a steady drop, hence supporting the model's dependability.

The statistically substantial gains highlight HTM-MDICE's efficacy in ethics teaching, especially its capacity to surpass both conventional techniques (e.g., Decision Tree, Petri Nets). The stability of the findings across folds and the low p-values suggest that these enhancements are strong and not artifacts of particular data splits. Although the paired *t*-tests on accuracy offer compelling proof of HTM-MDICE's superiority, other statistical tests could confirm its performance even more. For instance, paired *t*-tests on F1-score or MAE might evaluate significance in the context of class imbalance or prediction calibration. To compensate for possible non-normality in the accuracy variations, non-parametric tests like the Wilcoxon signed-rank test might be investigated. Section 5 will take into account these studies together with potential future paths to improve the deployment and assessment of the model.

While the paired t-test for accuracy was initially applied, we recognize that additional significance testing on other key metrics—namely *F1-score* and *MAE*—is crucial to offer a fuller understanding of HTM-MDICE's capabilities. In particular, *F1-score* is particularly relevant given the class imbalance in the dataset, while *MAE* provides insights into the model's calibration.

#### Significance testing for F1-score and MAE

Through paired t-tests for both F1-score and MAE, we examined whether HTM-MDICE performs better than the baseline models on these advantageous metrics. The F1-score is an important metric when classes are imbalanced, as it is helpful for balancing the precision-recall tradeoff—an important tradeoff in binary classification tasks, such as predicting engagement. MAE is an important metric for assessing the calibration of the model by evaluating the average absolute error between the predicted values and the true values.

The results of the paired *t*-tests on F1-score and MAE indicate that HTM-MDICE performs significantly better than the baseline models on both metrics, with p-values consistently below 0.05. For example, HTM-MDICE had an F1-score of 0.96, while the baseline models performed worse, such as BSA-ANN (0.88) and Petri Nets (0.80). Also, for MAE, HTM-MDICE outputted 0.12, while BSA-ANN and Petri Nets outputted 0.20 and 0.27, respectively. These results further indicate that HTM-MDICE provides better predictive accuracy and calibration, which is essential for making qualitatively reliable predictions in education contexts.

#### Non-parametric testing with Wilcoxon signed-rank test

In order to bolster our findings and help address deviations to distributional assumptions we also performed the Wilcoxon signed-rank test, a non-parametric alternative to the paired *t*-test. The Wilcoxon signed-rank test is a good option when data violates the normality assumption for parametric tests, such as the paired t-test. We applied the Wilcoxon signed-rank test both F1-score and mean absolute error (MAE) in order to further bolster that this wasn't just a violation of distributional assumptions and are truly differences in means.

The Wilcoxon signed-rank test also confirms findings from the paired t-tests, showing all *p*-values were significant (all *p* < 0.05), which further demonstrates the advancement of HTM-MDICE over baseline models. This analysis provides additional validation that the changes in both F1-score and MAE are a result of systematic differences in means (changes in system performance) and not random variation.

To provide a clear overview of the significance testing results, we have included updated tables summarizing the *p*-values for *F1-score, MAE*, and *accuracy*, both from the paired *t*-tests and the *Wilcoxon signed-rank tests*. These tables show that HTM-MDICE consistently outperforms the baseline models across all three metrics, with statistical significance across both parametric and non-parametric tests.

Table 9 (below) summarizes the *p*-values from the paired *t*-tests and *Wilcoxon signed-rank tests* for *F1-score* and *MAE*, further supporting the robustness of HTM-MDICE's performance.

**Table 9:** *P*-values from statistical significance testing.

**Metric**	**BSA-ANN**	**Decision tree**	**BPNN**	**Petri nets**
F1-score (Paired *t*-test)	0.002	0.0003	0.001	0.0001
MAE (Paired *t*-test)	0.001	0.0005	0.002	0.0001
F1-score (Wilcoxon)	0.003	0.0004	0.002	0.0002
MAE (Wilcoxon)	0.001	0.0006	0.003	0.0001

The results from both the paired *t*-tests and Wilcoxon signed-rank tests underscore the consistency and significance of HTM-MDICE's performance improvements over the baseline models.

## Discussion

With a validation accuracy of 97.5%, an F1-score of 0.96, and a MAE of 0.12 at the optimal early stopping patience of 5 (Section 4.1), the HTM-MDICE model demonstrated remarkable effectiveness in predicting student engagement and ideological understanding on the ethics dataset. Statistical analysis (Section 4.3, Table 7) revealed that HTM-MDICE significantly outperformed four prior methods, with accuracy gains ranging from 5.5% to 10.5% (relative to Petri Nets, *p* = 0.0001). HTM-MDICE's outstanding performance is credited to its Transformer-based design, which deftly catches complex temporal and contextual patterns within the ethical dataset, encompassing 68,200 instances, 1,000,000 numerical data points, and 500,000 behavioral logs. Unlike traditional methods like Decision Tree (88.5% accuracy) or Petri Nets (87.0%), which rely on simple feature representations, HTM-MDICE uses multi-head attention mechanisms to capture sequential linkages across situations, numerical data, and behavioral logs. HTM-MDICE benefits from its hierarchical temporal modeling, which is particularly good for the complex, time-sensitive components of student involvement and understanding. The high F1-score (0.96) shows good class imbalance management in the virtue domain (25,830 Label 0 occurrences against 2,415 Label 1 instances), thereby enabling accurate detection of strong engagement (Label 1), which is crucial for instructional purposes. These improvements' statistical significance—all *p* < 0.05—supports the idea that HTM-MDICE's benefits are not accidental. HTM-MDICE's success was greatly influenced by the preprocessing pipeline. Providing effective integration of different data sources, scenario texts were tokenized using a proprietary tokenizer, numerical data points were standardized to a [0, 1] range, and behavioral logs were embedded using a linear layer. Unlike methods like Decision Trees, which used simple bag-of-words encodings, or Petri Nets, which represented data as discrete states, this standardization let the Transformer design identify notable patterns across modalities. Performance on the minority class was enhanced by careful handling of class imbalance using weighted binary cross-entropy loss, which produced a high F1-score and a low false negative rate as seen in the confusion matrix (Figure 12).

### Ethical implications

The use of AI for predicting student engagement and ideological understanding introduces several ethical challenges, particularly concerning misclassification. False positives may lead to unnecessary interventions, while false negatives could result in students who need help being overlooked. To mitigate these issues, we propose the use of continuous monitoring, a human-in-the-loop approach, and transparent explainable AI techniques that enable educators to verify predictions before taking action. Furthermore, labeling students as having ‘low engagement' can lead to stigmatization, which is detrimental to their self-esteem and classroom dynamics. To address this, we advocate for careful communication of AI predictions in terms of ‘risk levels' rather than fixed labels and for fostering a growth mindset among students. Additionally, teachers should receive training on ethical AI interpretation to ensure that predictions are used to support students rather than assign blame.

While HTM-MDICE provides valuable insights into student engagement and ideological understanding, it is essential to recognize the ethical implications of misclassification, particularly for underrepresented groups. Misclassification can lead to unnecessary interventions, stigmatization, or lack of support, particularly for students from marginalized communities. To mitigate these risks, we advocate for human-in-the-loop decision-making, where AI predictions are used as supportive tools rather than definitive judgments. Moreover, models should be regularly tested for fairness and adjusted to ensure they do not disproportionately harm underrepresented students. Educators must also receive training to interpret AI predictions in context and understand the limitations of the model in predicting student engagement and performance.

Even though we used early stopping with patience = 5 to mitigate overfitting, it is suggested that, given the imbalance in the dataset, the 97.5% test accuracy found for both the HTM and MDICE models could also be indicative of overfitting in this case. Confirming the mitigating effect of early stopping, however, provided critically important stop gaps to ensure that validation loss did not assert itself as improvement. Future studies should explore further regularization strategies by investigating model dropout or even cross-validation to establish reliability and the empirical utility of the models across any unseen data.

The substantial computational demands associated with HTM-MDICE's Transformer-based architecture might hinder its practical applicability as the model is limited by the computational influence of smaller organizations that do not have access to robust resources. In the case that HTM-MDICE models demonstrated strong performance in practice, the level of computational effort could discourage\ smaller educational institutions and other smaller practitioners from applying such computational usefulness. Future work should look for ways to simply HTM-MDICE or leverage cloud-based tooling to reduce the computational burden on users, making institutional adoption easier for a wider range of educational practice.

### Future directions

Although this research examined HTM-MDICE's performance against several baseline models (BSA-ANN, Decision Tree, BPNN, and Petri Nets), we recognize the potential contribution of more recent deep learning methods, such as CNNs, advanced LSTMs, and hybrid attention models, in enhancing predictive validity and augmenting the interpretability of predictions. We selected baseline models, likewise, on the basis of their prior usage in the educational domain and compatibility with dataset features. Future research can build on this work, directing attention to these more recent models to evaluate their predictive success of student engagement and ideological understanding in multimodal education datasets.

In future studies, we plan to expand the baseline comparison by incorporating more contemporary deep learning models such as CNNs, advanced LSTMs, and hybrid attention models. These models may provide a deeper understanding of their effectiveness in multimodal data fusion and their impact on predictive performance in educational contexts.

While early stopping was employed to prevent overfitting, the potential for overfitting remains a concern, particularly with a highly imbalanced dataset. Future work should implement cross-validation, explore additional regularization techniques, and evaluate the model's performance on external datasets to confirm its generalizability.

To further evaluate the generalizability of HTM-MDICE, future work must apply the model to autonomous datasets obtained from multiple institutions, regions, and educational settings. This validation will guarantee that the model's predictions are not only accurate predicts within a dataset but also generalizable consistency and validity across different educational contexts. External validation will also provide evidence of the model's performance in consideration of different curricula, pedagogical styles, and cultural influences

### Ethical oversight and educator responsibility

While no identifiable personal data was used, categorizing students' moral and ethical reasoning inherently involves interpreting subjective responses, which could introduce ethical concerns. The risk of misclassification or oversimplification of complex ethical views may have unintended consequences. Therefore, it is crucial that educators use the predictions of the model as one tool among many, ensuring that AI-driven insights do not replace human judgment but instead augment it.

### Educator use of SHAP interpretability

In addition to the general use of SHAP for explaining model predictions, we have provided specific examples and visualizations of feature importance to help educators interpret the model's results. By analyzing which features (e.g., keywords in essays, frequency of participation) most impact predictions, educators can use this information to better understand student engagement. For instance, a prediction of ‘low engagement' may be influenced by a student's lack of participation in discussions or limited interaction with course materials, which can guide educators in offering targeted support.

### The effect of early stopping

Achieving an optimal balance between convergence and generalization was significantly dependent on using early stopping with a patience of 5 (38 epochs). With modest overfitting relative to patience = 7 (63 epochs, slight loss spike in Figure 14), the training and validation curves (Figures 9, 11, 12) show constant improvement from initial accuracies of about 50% to 97.5%. This setup allowed HTM-MDICE to completely tune its settings while avoiding the early termination observed with patience = 3 (17 epochs, 96.8% accuracy). Early Stopping improved robustness and computing efficiency, hence qualifying the model for practical use. Meticulously designed to handle the complexity of the dataset, the HTM-MDICE architecture had 12 Transformer layers, 8 attention heads, and a hidden dimension of 512. While dropout (0.1) lowered overfitting, positional encodings kept temporal correlations so the model could outperform more basic designs like BPNN (90.5%). Unlike CNN-based approaches, HTM-MDICE's attention mechanisms provided better contextual understanding, hence improving accuracy by 5.5%. Training stability was enhanced by the use of the AdamW optimizer with a learning rate of 2e-5 and a weight decay of 0.01. The use of HTM-MDICE in educational settings raises ethical questions. Misclassifying students as uninterested might lead to unfair treatment or lower opportunities, particularly for those who interact in less obvious ways.

While the early stopping with patience = 5 yielded optimal performance on the dataset used in this study, future research should investigate the impact of different early stopping strategies across diverse datasets. Sensitivity analysis on patience values will be crucial for assessing the robustness and generalizability of the model to other contexts.

### Limitations

Even though the performance metrics are encouraging, we must keep in mind that the increased accuracy, F1-score and MAE are all largely characteristics of the dataset, specifically class imbalance and the limited institutional and cultural context. They cannot necessarily be said to generalize to a dataset with different distributions or a different institutional context altogether.

Despite HTM-MDICE producing useful and significant results for predicting student engagement and ideological understanding, we must be careful to point out that what is considered ethical education will vary greatly and widely across cultures. The model is assuming and predicting behaviors of the student and teacher based on reporting information from a particular institution in China; thus, it is unknown how these findings will generalize to other educational systems and cultures. Future work should examine how ethical decision-making and ethical engagement vary across cultures, as well as how the model may be adapted to different educational contexts.

The dataset for this study was collected from a single institution (Shandong Sport University) in China, which limits the ability to generalize findings to different contexts, institutions, or regions. The ethical and engagement patterns identified within this dataset may not be similar to those of students from other educational systems and cultures, suggesting that external validation will be necessary for more diverse datasets. Further, ethical decision-making and engagement behavior can vary considerably across cultures, so future studies should investigate how these findings apply to other educational systems and cultural contexts.

The dataset also presented a significant class imbalance between the majority class (low engagement/understanding) and minority class (high engagement/understanding) which could lead to possible bias in the models' predictions. Weighted loss functions and oversampling approaches were used to address this, but overfitting and skewed predictions are still a concern, especially for the minority class. Future studies should incorporate additional methodology such as undersampling or class-balanced loss functions to further address this class imbalance.

While the use of SHAP for model interpretability is a step toward transparency, it is still limited by the model's complexity. Educators may face challenges in fully understanding or applying the model's predictions, especially when making decisions about at-risk students. Despite SHAP's ability to explain the contributions of different features to predictions, further research is needed to make HTM-MDICE's interpretability more accessible and actionable for educators. Simplifying the explanation process and providing more intuitive visualizations of the model's outputs could improve the usability of the model in practical educational settings.

### Practical considerations and computational efficiency

HTM-MDICE has demonstrated significant potential with encouraging results; however, the computational requirements may be prohibitive to smaller organizations. In future work, efforts should be made to simplify the model or consider cloud-based applications to improve the accessibility of this approach. Research efforts could also consider the utilization of model distillation techniques to produce model alternatives that reduce computational burden while preserving levels of predictive performance.

### Interpretability and educator use of SHAP

To ensure that HTM-MDICE's predictions are interpretable and actionable for educators, we used SHAP (SHapley Additive exPlanations) to explain each prediction for every student by assigning each feature (or predictor) a value that reflects its contribution to the model output. By visualizing these SHAP values, educators can see which features contributed the most to the predicted engagement or ideological understanding of a given student.

SHAP also provides a variety of visualizations to decipher the model predications into interpretable impact. If we consider the satisfied response (i.e., one of the model outputs) as a model prediction, the SHAP summary plot is a particularly useful visualization to show how each feature (e.g., essay keywords, frequency of participation, etc.) contributed to the model's several predictions across all students. In this visualization, each feature is ranked based on its importance, and each features' value (e.g., presence of “justice” or “equality”) is also reported alongside the feature's contribution to the model's prediction.

As an example of practice, a student predicted to have low engagement may be portrayed in the SHAP analysis (Figure 12) as exhibiting low involvement in online discussions and low duration of activities as two of the top features associated with this engagement prediction. The instructor can utilize this information to reveal areas in which the student may need more engagement or to bolster participation. In the case of a student indicated to have high engagement, SHAP values may show characteristics of frequent engagement and the use of complex moral reasoning terms such as “rights” and “justice” as contributing features.

This type of information allows instructors to understand not just the why behind a student's prediction of engagement but also the how and where to intervene.

Instructors can use SHAP visualizations in practice to:

- Distinguish students who are flagged for low engagement or low ideological understanding and understand exactly what behaviors or content may have contributed to that prediction.
- Plan appropriately targeted intervention, which may take the form of encouraging additional participation during discussions or focusing on moral reasoning within assignments.
- Integrate model outputs as findings within the context of the instructor's professional judgment and not fully rely on either or both AI predictions and their use in the prediction outputs.

## Conclusion

This study has demonstrated the effectiveness of the HTM-MDICE model, a Transformer-based approach designed to predict student engagement and ideological understanding within the context of ethics education. By achieving a validation accuracy of 97.5%, an F1-score of 0.96, and an MAE of 0.12 at an early stopping patience of 5, HTM-MDICE significantly outperformed four prior methods, with accuracy improvements ranging from 5.5% to 10.5% and all *p*-values below 0.05, as confirmed by paired *t*-tests. HTM-MDICE's development and a comprehensive evaluation process employing statistical analysis, qualitative input, and quantitative measures mark a major advance in the area of educational predictive modeling. Its robust preprocessing pipeline, decent early stopping approach, and Transformer architecture help the model to perform well. These taken together enable it to manage complex, multi-modal data and class imbalance in the virtue domain. These findings provide teachers with a tool to enable them to identify and promote student participation, hence contributing to the expanding field of personalized education.

Prospective modifications may enhance the utility of HTM-MDICE and amplify its impact. Incorporating diverse data types—such as reflective diaries, student self-reports, and physiological indicators like eye-tracking—could enhance the model's comprehension of intricate engagement patterns, hence reducing the likelihood of misclassifying pupils with minimal involvement. Employing methodologies such as feature importance analysis or explainable AI to enhance the comprehensibility of forecasts will augment transparency and address students' demands for greater insight into their engagement rates. Performance may be enhanced by rectifying technical issues associated with the stacking ensemble approach, particularly those related to its integration with scikit-learn. Smaller schools with constrained computing resources may find it more feasible to utilize the model if simplified variants are developed. Ultimately, fairness audits will be essential to address ethical dilemmas and ensure that projections serve as aids rather than definitive conclusions. This is essential for accountable implementation. Adhering to these directives will enable HTM-MDICE to further develop as a robust and ethical instrument for enhancing individual learning and fostering greater interest and comprehension in ethics and related fields.

## Data Availability

The original contributions presented in the study are included in the article/supplementary material, further inquiries can be directed to the corresponding author.
